# Worldwide Prevalence of Intimate Partner Violence in Pregnancy. A Systematic Review and Meta-Analysis

**DOI:** 10.3389/fpubh.2021.738459

**Published:** 2021-08-30

**Authors:** Rosario M. Román-Gálvez, Sandra Martín-Peláez, Borja M. Fernández-Félix, Javier Zamora, Khalid S. Khan, Aurora Bueno-Cavanillas

**Affiliations:** ^1^Departamento de Enfermería, Facultad de Ciencias de la Salud, Universidad de Granada, Granada, Spain; ^2^Unidad Asistencial Alhama de Granada, Servicio Andaluz de Salud, Granada, Spain; ^3^Departamento de Medicina Preventiva y Salud Pública, Facultad de Medicina, Universidad de Granada, Granada, Spain; ^4^Instituto de Investigación Biosanitaria de Granada IBS, Granada, Spain; ^5^Clinical Biostatistics Unit, Hospital Ramón y Cajal, Instituto Ramón y Cajal de Investigación Sanitaria (IRYCIS), Madrid, Spain; ^6^Centro de Investigación Biomédica en Red (CIBER) Epidemiología y Salud Pública, Instituto de Salud Carlos III, Madrid, Spain

**Keywords:** pregnancy, intimate partner violence, domestic violence, prenatal care, prevalence

## Abstract

**Background:** Intimate partner violence (IPV) affects outcomes of mothers and their offspring. This systematic review collated the worldwide literature on the prevalence rates of different types of IPV in pregnancy.

**Methods:** Two reviewers independently identified cross sectional and cohort studies of IPV prevalence in pregnancy in online databases (PubMed, WOS and Scopus), selected and extracted data [participants' country, study quality, measurement tool (validation and purpose) and rates of IPV in pregnancy]. We considered a high quality study if it had a prospective design, an adequate sampling method, a sample size estimation, a response rate > 90%, a contemporary ascertainment of IPV in the index pregnancy, and a well-developed detailed IPV tool. We performed random effects meta-analysis and explored reasons for heterogeneity of rates.

**Results:** One hundred fifty-five studies were included, of which 44 (28%) met two-thirds of the quality criteria. Worldwide prevalence of physical (126 studies, 220,462 participants), psychological (113 studies, 189,630 participants) and sexual (98 studies, 155,324 participants) IPV in pregnancy was 9.2% (95% CI 7.7–11.1%, I^2^ 95.9%), 18.7% (15.1–22.9%, I^2^ 98.2%), 5.5% (4.0–7.5%, I^2^ 93.4%), respectively. Where several types of IPV were reported combined, the prevalence of any kind of IPV (118 studies, 124,838 participants) was 25.0% (20.3, 30.5%, I^2^ 98.6%). IPV rates varied within and between continents, being the highest in Africa and the lowest in Europe (*p* < 0.001). Rates also varied according to measurement purpose, being higher for diagnosis than for screening, in physical (*p* = 0.022) and sexual (*p* = 0.014) IPV.

**Conclusions:** IPV prevalence in pregnancy varies across countries, with one-quarter of mothers exposed on average globally. Routine systematic antenatal detection should be applied worldwide.

**Systematic Review Registration:** identifier: CRD42020176131.

## Introduction

Intimate partner violence (IPV), defined as physical violence, psychological assault and sexual abuse (including coercive tactics) by a current or former intimate partner (1) is an avoidable global public health problem. IPV in pregnancy, despite being more common than other obstetric problems like preeclampsia or gestational diabetes (2) remains a neglected condition. It is associated with adverse pregnancy outcomes including increased risk of human immunodeficiency virus infection (3) perinatal depression (4) uterine rupture, hemorrhage, maternal death (5) prematurity, low birth weight, newborns small for gestational age (6) stillbirth (7) insufficient weight gain in pregnancy (8) and reduced levels of breastfeeding (9).

IPV prevalence in pregnancy is reported to vary according to the definition used (1) the measurement strategy (10, 11) and the socio-cultural context of the population studied (2, 12). These factors make comparison between individually reported rates difficult. An umbrella review of collated summaries of the published worldwide and country IPV prevalence data in pregnancy (13) showed that the existing evidence syntheses did not capture the totality of the disease burden, they did not always specify the gestational time point of IPV evaluation, and sometimes the IPV and domestic violence concepts were mixed up, leaving ambiguity. When assessed for quality, the existing reviews showed possible bias in study selection, general failure in addressing heterogeneity, and a narrow geographical coverage, focused on low-income countries (13). A robust updated systematic review of IPV prevalence in pregnancy is required to provide underpinning evidence for antenatal care policies.

Our objective was to synthesize worldwide prevalence data concerning physical, psychological, and sexual IPV in pregnancy and to explore reasons for variations in rates.

## Methods

The systematic review was carried out following prospective registration (PROSPERO ID: CRD42020176131) and reported according to the PRISMA statement (14).

### Literature Search and Selection

Searches were conducted in PubMed, WOS and Scopus databases from inception to January 2021. The following search string combining medical subject headings (MeSH) and free text terms was used ((“Intimate Partner Violence” AND (“Pregnancy” OR “Pregnant Women” OR “Prenatal Care”)) AND “Prevalence”). In addition, relevant citations were scanning from reference lists of the selected articles. We contacted authors of key relevant citations by email for studies known to them of the subject. Two reviewers (RMRG and SMP) independently selected citation and studies meeting the following criteria: cross-sectional or cohort study design and a tool was used to estimate the rate of IPV in pregnancy. No language restrictions were applied.

### Data Extraction and Quality Assessment

Two reviewers (RMR-G and SM-P) selected studies and extracted data independently, using a piloted form, to capture study, participant, and tool characteristics.

Adapting existing quality assessment tools (15, 16), we considered the following items and coding for evaluation of risk of bias: (a) a prospective design (yes, no, not reported or not evaluable); (b) *a priori* sample size estimation for precision of IPV rate (yes, no, not reported); (c) appropriate methods to capture a representative sample based on use of random or consecutive sampling, demonstration of similarity of sample characteristics to those of the population, (yes if any of these features were present, no, not reported or not evaluable); (d) contemporaneous ascertainment of IPV in the index pregnancy (yes, no, not reported); (e) use of a well-developed, detailed IPV tool with a published reference concerning its performance applied to the whole sample or to screen positive women, but not assessment by screening tools only [yes, no, not reported or not evaluable, using a published source (17); (f) relevance of IPV tool to local population either through development of the tool in their language or, if not originally developed in the same language, with translation and back translation, ideally with robust translated tool performance evaluations (yes, no, not reported or not evaluable); and (g) response rate over 90% (yes, no, not reported or not evaluable). We considered a study to be of high quality with respect to estimation of representative and unbiased IPV rates if it met at least 5 and low if met <3 of the criteria above. We calculated inter-reviewer agreement for data extraction concerning quality using kappa index to determine reliability (18). In cases of disagreement we used consensus and arbitration by a third reviewer (ABC).

We extracted data concerning rates for each IPV type separately and combined. Numerator data concerning physical violence, psychological assault and sexual abuse were classified as reported by the authors of the selected papers.

### Data Synthesis

Prevalence rates of IPV were estimated with 95% confidence intervals (CI) overall using a multilevel random effects logistic regression model with random intercepts for each study. Statistical heterogeneity was assessed graphically, using “forest” plots, and statistically, through the percentage of variation across studies (I squared statistic) and using the τ^2^ statistic. Sources of heterogeneity were evaluated by subgroups analysis, that were performed for geographical area (i.e., continent), type of instrument (validated, ad-hoc or unreported) and measurement purpose (screening, diagnosis or unreported). We used meta-regression models to evaluate these associations as well as to assess the association with year of study publication. Funnel plots and Egger's tests were used to evaluate publication bias and small study effects, given extreme values of proportions (near 0 or 1) we used Freeman Tukey arcsin transformation to stabilize variances for this analysis (18–20). All statistical analyses were performed using Stata statistical software version 16.0 (21).

## Results

**Figure 1:**
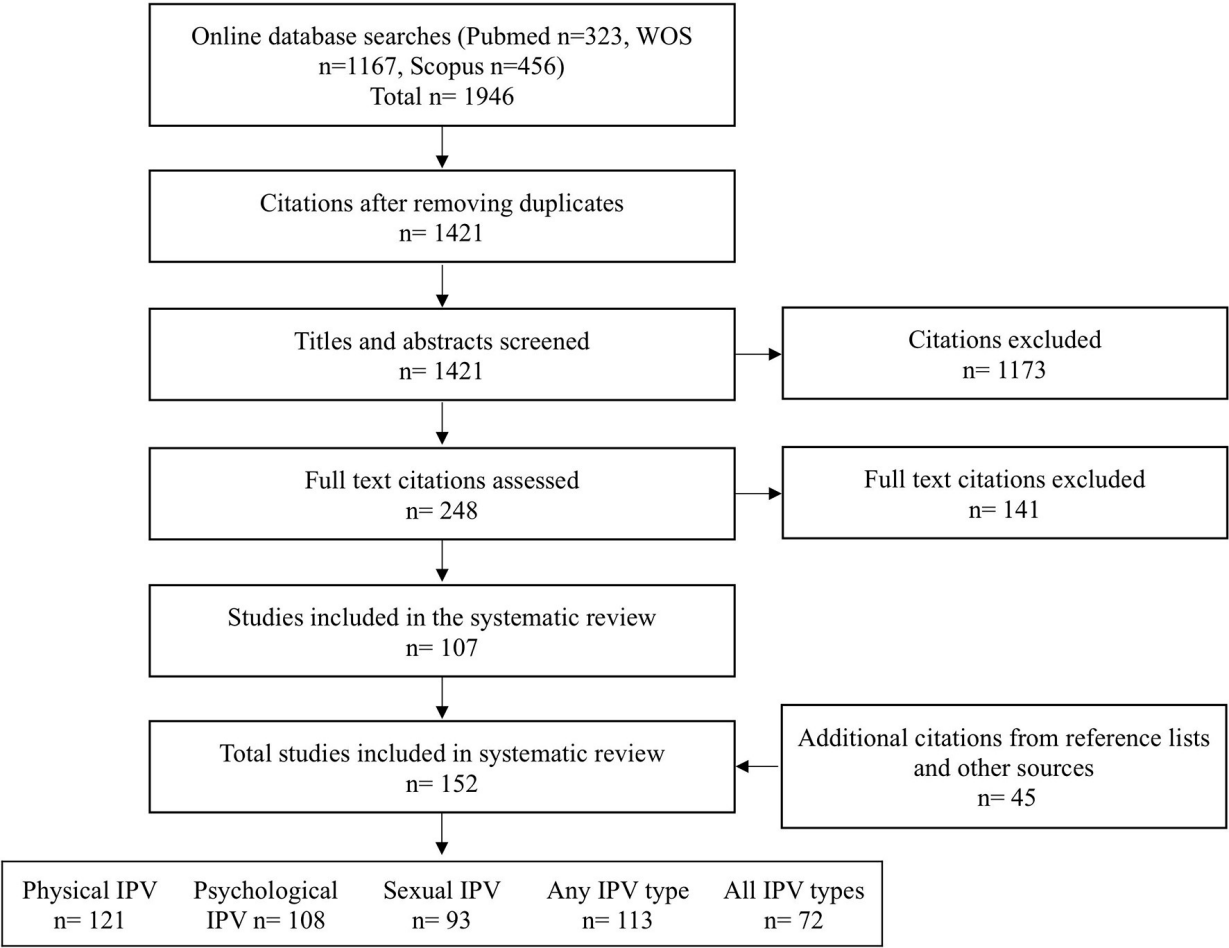
Flowchart systematic review of intimate partner violence during pregnancy.

The electronic search yielded a total of 1,946 citations (Figure 1). After elimination of duplicates, 1,421 titles and abstracts were examined. Of these, 248 were found to be potentially relevant and their full articles were obtained. After reviewing these, 141 articles were excluded (Supplementary Table 1). The reference lists of the remaining 107 articles revealed 45 further citations. Finally, 152 articles (157 studies) met the inclusion criteria, providing data on 322,572 participants. Physical, psychological and sexual IPVs were all reported in 72 (45.9%) studies; only one type of IPV, commonly physical, was reported in 32 (20.5%) studies. The details of studies included in the systematic review on IPV prevalence in pregnancy are given in Supplementary Table 2. Study quality assessment (Supplementary Figure 1) revealed deficiencies in many areas of methodology, particularly those related to measurement tool, response rate and a priori sample size estimation. Overall, at least 5/7 quality criteria were met by 44 (28.0%) studies, 3–4/7 criteria by 92 (58.6%) studies, and <3/7 criteria by 21 (13.4%) studies. Inter-reviewer agreement concerning quality items was median Kappa 0.8 (range 0.6–1).

**Figure 2:**
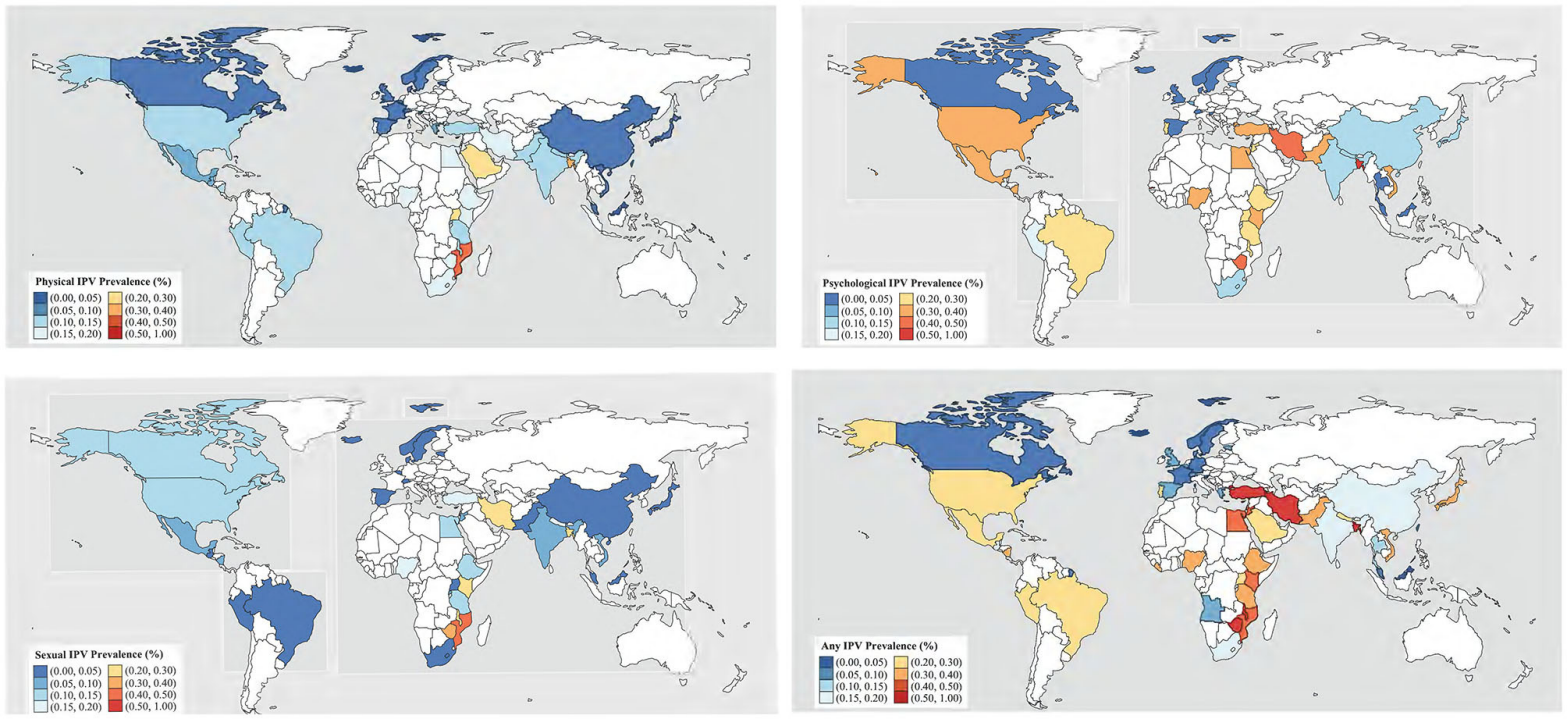
World-wide distribution of intimate partner violence (IPV) types.

The data on IPV prevalence in pregnancy is summarized in Table 1. Worldwide prevalence of physical (126 studies, 220,462 participants), psychological (113 studies, 189,630 participants) and sexual (98 studies, 155,324 participants) IPV in pregnancy was 9.2% (95% CI 7.7–11.1), 18.7% (95% CI 15.1–22.9), 5.5% (95% CI 4.0–7.5), respectively. Where several types of IPV were reported combined, the prevalence of any kind of IPV (118 studies, 124,838 participants) was 25.0% (95% CI 20.3–30.5). There was heterogeneity. The rates did not vary according to year of publication (Supplementary Figure 2). The retrieved data were from 52 countries. Figure 2 shows the worldwide prevalence rates by country concerning different types of IPV in pregnancy. Supplementary Table 3 gives the underlying data.

**Table 1:** Meta-analysis by type of intimate partner violence (IPV) during pregnancy.

**IPV**	**Studies**	***n***	***N***	**Rate**	***I*^**2**^ (%)**	***P*-value heterog**	**Tau^**2**^**
Physical	126	12,801	220,132	9.3 (7.7, 11.1)	95.9	0.000	1.31
Psychological	113	29,446	189,630	18.7 (15.1, 22.9)	98.2	0.000	1.87
Sexual	98	7,585	155,324	5.5 (4.0, 7.5)	93.4	0.000	2.67
Any	118	24,779	124,558	25.2 (20.4, 30.7)	98.6	0.000	2.25

*n = IPV events. N = group size*.

The prevalence rates of physical IPV in pregnancy ranged from 0.7% (22) to 55.1% (23) in 126 studies including 220,462 pregnant women. Worldwide prevalence rate of physical IPV in pregnancy was 9.2% (95% CI 7.4–11.1). Prevalence figures were higher in Africa (16.3%, 95% CI 13.5–19.6) than in Asia (9.0%, 95% CI 6.5–12.3), North (9.0%, 95% CI 5.3–15.1) or South America (9.8%, 95% CI 7.3–13.0). There were only two studies from the Oceania region. The lowest prevalence was reported for Europe (2.1%, 95% CI 1.3–3.4). There were no differences according to the use or not of a validated tool, but prevalence figures were higher when the purpose of measurement was diagnosis instead of screening (Table 2).

**Table 2:** Worldwide prevalence data for intimate partner violence (IPV) in pregnancy according to type of violence.

**IPV type**	**Continent/IPV tool/Measurement purpose**		**Studies**	***N***	***n***	**Rate (%)**	***I*^**2**^ (%)**	**Tau^**2**^**	***P*-value**
Physical	Continent	Africa	38	25,146	4,069	16.3 (13.5, 19.6)	95.26	0.49	<0.001
		Asia	43	127,091	5,836	9.0 (6.5, 12.3)	95.81	1.32	
		Europe	17	35,540	671	2.1 (1.3, 3.4)	90.17	0.98	
		North America	11	11,204	538	9.0 (5.3, 15.1)	91.55	0.92	
		South America	15	21,052	1,630	9.8 (7.3, 13.0)	93.72	0.37	
		Oceania	2	429	82	19.1 (15.7, 23.1)	..	..	
	IPV tool	Validated	84	64,048	6,511	10.0 (8.0, 12.4)	95.91	1.26	0.517
		*Ad hoc*	23	28,630	2,308	8.3 (5.8, 11.8)	95.78	0.89	
		Unreported	19	127,784	4,007	7.6 (4.3, 13.2)	95.52	1.82	
	Measurement purpose	Screening	18	13,119	820	5.8 (3.4, 9.6)	93.07	1.33	0.022
		Diagnosis	61	48,293	5,431	11.6 (9.0, 14.8)	96.43	1.22	
		Unreported	46	158,242	6,464	8.0 (6.0, 10.8)	95.68	1.20	
Psychological	Continent	Africa	35	22,784	6,108	25.2 (18.9, 32.6)	98.15	1.19	<0.001
		Asia	40	121,717	18,262	19.3 (13.7, 26.5)	98.34	1.72	
		Europe	15	23,073	1,269	4.2 (2.4, 7.4)	95.64	1.29	
		North America	8	9,680	840	28.6 (10.1, 58.7)	97.32	3.32	
		South America	13	11,947	2,795	23.4 (19.1, 28.3)	94.81	0.21	
		Oceania	2	429	172	40.1 (35.6, 44.8)	0.00	0.00	
	IPV tool	Validated	78	64,487	10,966	18.0 (13.8, 29.1)	98.06	1.99	0.707
		*Ad hoc*	19	25,276	4,609	18.3 (11.4, 28.1)	98.35	1.51	
		Unreported	16	99,867	13,871	23.1 (13.7, 36.2)	98.40	1.67	
	Measurement purpose	Screening	13	13,217	1,442	11.9 (7.4, 18.8)	97.09	0.94	0.270
		Diagnosis	61	49,442	8,966	18.8 (13.7, 25.2)	98.07	2.24	
		Unreported	39	126,971	19,038	21.5 (15.6, 28.7)	98.37	1.50	
Sexual IPV	Continent	Africa	35	23,740	3,918	12.4 (8.6, 17.4)	96.90	1.43	<0.001
		Asia	30	27,350	2,706	6.6 (4.1, 10.7)	94.60	2.00	
		Europe	13	91,376	342	0.5 (0.3, 0.9)	66.99	0.98	
		North America	5	942	74	8.9 (3.8, 19.4)	86.91	0.96	
		South America	13	11,487	437	2.7 (1.6, 4.4)	87.01	0.76	
		Oceania	2	429	108	25.2 (21.3, 29.5)	0.00	0.00	
	IPV tool	Validated	71	130,733	4,968	5.2 (3.5, 7.6)	92.50	3.02	0.708
		*Ad hoc*	18	21,309	1,993	5.9 (3.6, 9.6)	94.73	1.23	
		Unreported	9	3,282	624	8.2 (2.9, 21.4)	94.29	2.73	
	Measurement purpose	Screening	12	6,562	144	1.6 (0.8, 3.4)	78.41	1.49	0.014
		Diagnosis	54	122,111	4,664	6.4 (4.0, 9.9)	93.95	3.14	
		Unreported	32	26,651	2,777	6.8 (4.4, 10.3)	94.76	1.63	
Any IPV^*^	Continent	Africa	38	23,380	8,863	36.1 (27.7, 45.4)	98.76	1.46	<0.001
		Asia	37	40,820	9,399	32.1 (22.7, 43.2)	98.85	2.17	
		Europe	18	34,964	1,668	5.1 (3.4, 7.5)	95.00	0.78	
		North America	9	11,615	1,078	20.4 (6.9, 47.1)	96.17	3.58	
		South America	14	13,630	3,587	25.6 (21.1, 30.7)	95.19	0.22	
		Oceania	2	429	215	50.1 (45.4, 54.8)	0.00	0.00	
	IPV tool	Validated	77	60,663	14,263	26.2 (20.0, 33.6)	98.57	2.46	0.694
		*Ad hoc*	23	28,451	5,892	20.9 (13.8, 30.2)	98.67	1.46	
		Not reported	18	35,724	4,655	25.7 (14.8, 41.0)	98.52	2.24	
	Measurement purpose	Screening	21	18,843	2,845	15.0 (9.8, 22.3)	97.33	1.26	0.075
		Diagnosis	53	40,305	10,740	29.3 (21.6, 38.4)	98.67	2.27	
		Unknown	44	65,690	11,225	25.9 (18.0, 35.6)	98.78	2.42	

*n = IPV events. N = group size.*

* *Any of the IPV types (physical, psychological or sexual) combined*.

The prevalence rates of psychological IPV in pregnancy ranged from 0.4% (24) to 79.8% (25) in 113 studies including 189,630 women. Global prevalence rate of psychological IPV in pregnancy was (18.7%, 95% CI 15.15–22.9). Prevalence figures were higher in North América (28.6%, 95% CI 10.1–58.7) than in Africa (25.2%, 95% CI 18.9–32.6), South America (23.4%, 95% CI 19.1–28.3) or Asia (19.3%, 95% CI 13.7–26.5). The lowest prevalence was reported for Europe (4.2%, 95% CI 2.4–7.4). There were no differences according to the use or not of a validated tool, neither according to the purpose of measurement (Table 2).

The prevalence rates of sexual IPV in pregnancy ranged from 0.0% (26) to 45.6% (27) in 98 studies including 155,324 women. Global prevalence rate of sexual IPV in pregnancy was (5.5%, 95% CI 40–7.5). Prevalence figures were higher in Africa (12.4%, 95% CI 8.6–17.4) than in North America (8.9%, 95% CI 3.8–19.4), Asia (6.6%, 95% CI 4.1–10.7) or South America (2.7%, 95% CI 1.6–4.4). The lowest prevalence was reported for Europe (0.5%, 95% CI 0.3–0.9). There were no differences according to the use or not of a validated tool, but prevalence figures were higher when the purpose of measurement was diagnosis instead of screening (Table 2).

The prevalence rates of any type IPV in pregnancy ranged from 1.8% (28) to 99.5% (29) in 118 studies including 124,838 women. Global prevalence rate of any type IPV in pregnancy was 25.0% (95% CI 20.4–30.7). Prevalence figures were higher in Africa (36.1%, 95% CI 27.7–45.4) than in Asia (32.1%, 95% CI 22.7–43.2), South (25.6%, 95% CI 21.1–30.7) or North America (20.4%, 95% CI 6.9–47.1). The lowest prevalence was reported for Europe (5.1%, 95% CI 3.4–7.5). There were no differences according to the use or not of a validated tool, neither according to the scope of the study, in spite of that the prevalence rate was twice for diagnosis than for screening studies (Table 2).

For all IPVs we found a significant small studies effect (Supplementary Figure 3). Smaller studies show higher prevalence estimates.

## Discussion

In this evidence synthesis, IPV prevalence in pregnancy was found to be variable, with one-quarter of mothers exposed to violence. The retrieved data from over 50 countries, showed that overall one in ten mothers were exposed to physical IPV, one in five to psychological IPV and one in twenty to sexual IPV on average. IPV prevalence was heterogenous within and between continents. The geographical differences showed that IPV rates were the highest in Africa (except for psychological IPV which was higher in North America) and the lowest in Europe. In the East of Africa and Southeast of Asia the rates are twice or more as high as the rest. There were no reliable data from the Oceania region.

This review have strengths and some limitations. We conducted this review with a rigorous methodology and reported it transparently (14). A prospective review protocol was used, and an organized effort made to identify all the available evidence without any language restriction. Most of the include studies were published from 2013 onwards. The population demographics are unlikely to have undergone major changes over this period, making the findings relevant to the current time period. However, from the funnel plot analyses we should beware that publication bias may inflate the estimated prevalence of IPVs. We did not include studies from conflict areas and those that restricted to women in specific pregnancy risk groups, such as HIV infection or adolescents. Thus, our findings are representative of overall IPV rates among pregnancies that routinely book for antenatal care.

The primary studies included in the review were generally poor in methodological quality. For example, appropriate methods to capture a representative sample lacked among two in five studies and the response rate was below 90% among two in three studies. A well-developed, detailed IPV tool was used only by under a third of the included studies. By independently synthesizing each type of IPV we aimed to aggregate data within clearly defined categories. A random effects meta-analysis provided conservative estimates of precision, a feature particularly suitable when there is heterogeneity, as in our review. Both the quality and the rates of IPV varied among the included primary studies and we comprehensively explored the reasons for these variations. Our findings concerning different types of IPV are subject to correct classification by authors. For example, in psychological IPV, verbal and emotional violence were mixed together in some studies (23, 30–32) but not in others (33–35). We did not find any significant effect for the use of a validated measurement tool in explaining heterogeneity, but we did find that the geographical area and the measurement purpose (screening vs. diagnosis among studies of physical and sexual IPV) explained variation partially. Unexplained heterogeneity is a common unavoidable feature of meta-analyses. This review represents the best available evidence summary of the global estimates of IPV prevalence in pregnancy at the time of writing.

There is a large number of countries lacking information. We have included only prevalence studies, excluding data from administrative or general surveys, which generally provide lower prevalences (36–39) related to IPV in pregnancy. Recently the WHO reported that 1 in 3 women, around 736 million, are subjected to physical or sexual violence by an intimate partner or sexual violence from a non-partner (40). Our results confirm this information, adding concrete data regarding women's reproductive life period with respect to pregnancy. The information on the observed rates of IPV across pregnancy have implications for provision of health and social services. Policy makers need to focus on systematic screening with provision of improved security and social care to women at IPV risk.

Pregnancy provides a window for IPV detection, therefore routine antenatal screening should be the norm rather than the exception. Although the observed variations in geographical distribution may be related to the definitions used, study characteristics or study quality, the main reason could be the differences in socio-cultural acceptation of violence against women, and thus, IPV. Both, the normalization of IPV and its sociocultural stigmatization of women exposed to IPV make its detection difficult (41). Pregnancy could be used to inform women that violence should not be accepted (42). Since IPV tends to increase in frequency, intensity and impact from the moment that it appears, any solution for IPV depends on its early detection. Finding out the frequency of the different types of IPV and the global extent of this problem is the first step in its resolution.

Differences in prevalence found by different authors in the same country is not readily appreciated in the prevalence maps. For the same country, taking Ethiopia as an example, Gebrezgi et al., provided a prevalence of any type of IPV of 98% in the Tigrai (43) area, whereas Gebreslasie et al., showed a prevalence of 7.3% (44). In another example in Kenya, the 67% prevalence of any type of violence reported by Owaka et al. (45), was almost duplicate of the 37% rate provided by Makayoto et al. (46). In yet another example in Turkey Yanikkerem et al. (29) found prevalences higher than 90% in Manis, Cengiz, whereas prevalences were lower than 3% in Istanbul (47). Our prevalence maps make it possible to identify large geographic areas for which epidemiological data are not available, highlighting Russia, Kzajistan, Mongolia, all of North Africa, Australia, Argentina, Chile, Colombia, Venezuela, Bolivia, etc. Thus, there are significant gaps in the knowledge of the prevalence of IPV in pregnancy worldwide. Future research must pay close attention to the study design and to the use of validated measurement tools for validity and comparability of the results across studies and regions. Such efforts will need funding agencies to be willing to support broader and more systems oriented approach.

## Conclusion

Overall, at least one in four pregnant women were exposed to one or another type of IPV in the worldwide literature. There were geographical differences. In the East of Africa and Southeast of Asia the figures are twice or more as high as the rest. Routine systematic IPV detection programs should introduced in antenatal care, a period of life when women at risk are likely to come in contact with health services.

## Data Availability Statement

The raw data supporting the conclusions of this article will be made available by the authors, without undue reservation.

## Author Contributions

RMR-G and SM-P were responsible for searching and selecting reviews, data extraction, and reviewed and provided input into the manuscript. KSK and AB-C planned the study design and the analysis and coordinated the analysis and writing of the manuscript. BMF-F and JZ maintained the database and did the analysis. All authors contributed intellectually to the work.

## Patient and Public Involvement

A patient representative was involved throughout the review, participated in review management meetings, and contributed to the evaluation of topic importance, interpretation of results and the generation of recommendations.

## Conflict of Interest

The authors declare that the research was conducted in the absence of any commercial or financial relationships that could be construed as a potential conflict of interest.

## Publisher's Note

All claims expressed in this article are solely those of the authors and do not necessarily represent those of their affiliated organizations, or those of the publisher, the editors and the reviewers. Any product that may be evaluated in this article, or claim that may be made by its manufacturer, is not guaranteed or endorsed by the publisher.
