# Supplementary material for: Worldwide Prevalence of Intimate Partner Violence in Pregnancy. A Systematic Review and Meta-Analysis
Source: Front Public Health. 2021 Aug 30;9:738459. doi: 10.3389/fpubh.2021.738459 (PMC8435609; doi:10.3389/fpubh.2021.738459)

***Supplementary material***

**Worldwide prevalence of intimate partner violence in pregnancy. A systematic review and meta-analysis**

**Table of contents**

Tables

Table S1. List of excluded studies ............................................................................................................3-5

Table S2. Characteristics of the selected studies......................................................................................6-29

Table S3. Intimate partner violence during pregnancy by country........................................................30-33

Figures

Figure S1. Studies meeting each quality item..............................................................................................34

Figure S2. Meta-regressions acording to publication year....................................................................35-36

Figure S3. Funnel plots and Egger’s tests for publication bias and small study effects..............................37

**Table S1. List of excluded studies**

|  | **Authors, year** | **DOI** | **Reason for exclusion*** |
| --- | --- | --- | --- |
| 1 | Rodrigues *et al*., 2008 | 10.1016 / j.ajog.2007.05.015 | A |
| 2 | Bernstein *et al*., 2016 | 10.1136 / bmjopen-2016-011999 | A |
| 3 | Certain *et al*., 2008 | 10.1111 / j.1552-6909.2007.00200.x | A |
| 4 | Clarke *et al*., 2019 | 10.1136 / bmjopen-2018-027541 | A |
| 5 | Salazar-Pousada *et al*., D. 2012 | 10.1016 / j.ijgo.2012.03.043 | B |
| 6 | Barcelona de Mendoza *et al*., 2018 | [10.1177 / 0886260515613346](https://dx.doi.org/10.1177%2F0886260515613346) | B |
| 7 | Hassan *et al*., 2014 | 10.1016 / j.puhe.2013.11.007 | B |
| 8 | Antai *et al*., 2012 | 10.1186 / 1471-2393-12-128 | C |
| 9 | Antunes Nunes *et al*., 2011 | 10.1093 / eurpub / ckp241 | A |
| 10 | Alhusen *et al*., 2018 | 10.1089/jwh.2017.6322 | C |
| 11 | Calderón *et al*., 2008 | [10.1016 / j.amepre.2007.09.029](https://doi.org/10.1016/j.amepre.2007.09.029) | D |
| 12 | Barnet *et al*., 2019 | [10.1136 / bmjopen-2017-018277](https://doi.org/10.1136/bmjopen-2017-018277) | E |
| 13 | Ahmed *et al*., 2006 | 10.2105 / AJPH.2005.066316 | F |
| 14 | Azad *et al*., 2019 | 10.1371 / journal.pone.0215735 | F |
| 15 | Azevedo *et al*., 2013 | 10.1590 / 0102-311x00161111 | F |
| 16 | Carneiro *et al*., 2016 | [10.1590/1980-5497201600020003](https://doi.org/10.1590/1980-5497201600020003) | G |
| 17 | Chen *et al*., 2017 | 10.1016/j.jnma.2017.06.017 | H |
| 18 | Barrios *et al*., 2015 | 10.1371 / journal.pone.0116609 | F |
| 19 | Beydoun *et al*., 2010 | 10.1016/j.socscimed.2011.01.006 | C |
| 20 | Bohn *et al*., 2004 | 10.1177/ 0884217504269009 | E |
| 21 | Brittain *et al*., 2015 | 10.1111/ppe.12216 | B F |
| 22 | Charles *et al*., 2007 | 10.1007/s10896-007-9112-0 | C |
| 23 | Cheng *et al*., 2015 | 10.1097/AOG.0000000000000609 | F |
| 24 | Chibber *et al*., 2014 | 10.1016 / j.whi.2013.10.007 | B |
| 25 | Cook *et al*., 2008 | 10.1258/jrsm.2008.080002 | E |
| 26 | Costa *et al*., 2017 | 10.1590/0102-311X00078515 | A |
| 27 | Cripe *et al*., 2010 | 10.1177/0886260509354517 | E |
| 28 | Dennis *et al*., 2013 | 10.1177/1077801213487057 | F |
| 29 | Doi *et al*., 2019 | [10.3389/fpubh.2019.00043](https://doi.org/10.3389/fpubh.2019.00043) | F |
| 30 | Durand *et al*., 2007 | [10.1590/S1415-790X2007000300003](https://doi.org/10.1590/S1415-790X2007000300003) | A C |
| 31 | Escriba-Agüir *et al*., 2013 | 10.1111/1471-0528.12051 | G |
| 32 | Ezechi, 2009 | 10.1007/s00404-009-0956-9 | B |
| 33 | Fawole *et al*., 2008 | 10.1111/j.1479-828X.2008.00868.x | A |
| 34 | Feder *et al*., 2018 | 10.1089/jwh.2017.6599 | E |
| 35 | Field *et al*., 2018 | 10.1186/s12905-018-0612-2 | F |
| 36 | Finnbogadottir *et al*., 2016 | 10.1186/s12884-016-1122-6 | G |
| 37 | Fisher *et al*., 2013 | 10.1093/inthealth/ihs012 | F |
| 38 | Fisher *et al*., 2018 | 10.1136 / bmjopen-2018-023539 | F |
| 39 | Flynn *et al*., 2007 | 10.1007/s00737-007-0188-6 | F |
| 40 | Gartland *et al*., 2011 | 10.1007/s10995-010-0638-z | F |
| 41 | Gaudard e Silva de Oliveira *et al*., 2017 | 10.1007/s00737-016-0705-6 | D |
| 42 | Gavin *et al*., 2011 | 10.1080/03630242.2011.606355 | I |
| 43 | Gee *et al*., 2009 | 10.1016/j.ajog.2009.04.048 | F |
| 44 | Glander *et al*., 1998 | [10.1016/S0029-7844(98)00089-1](https://doi.org/10.1016/S0029-7844(98)00089-1) | F |
| 45 | Groves *et al*., 2015 | 10.1177/0886260511425247 | E |
| 46 | Habtamu Belete *et al*., 2019 | 10.1186/s13104-019-4717-y | F |
| 47 | Hall *et al*., 2016 | 10.1371/journal.pone.0165621 | F |
| 48 | Harrison *et al*., 2008 | [10.1353/hpu.2008.0003](https://doi.org/10.1353/hpu.2008.0003) | F |
| 49 | Hartley *et al*., 2011 | 10.1001/archpsyc.61.9.946 | D |
| 50 | Hayati *et al*., 2011 | 10.1186/1472-6874-11-52 | F |
| 51 | Hayes *et al*., 2010. | 10.1007/s10995-009-0504-z | C |
| 52 | Heaman MI, 2005 | 10.1177/ 0884217505281906 | E |
| 53 | Henriques *et al*., 2015 | 10.1590 / 0102-311X00030215 | G |
| 54 | Holliday *et al*., 2017 | 10.1089/jwh.2016.5996 | F |
| 55 | Inami *et al*., 2010 | 10.1111/j.1742-7924.2010.00140.x | F |
| 56 | Johnson *et al*., 1997 | .. | F |
| 57 | Kastello *et al*., 2016 | 10.1007/s00737-015-0594-0 | D |
| 58 | Kingston *et al*., 2016 | 10.1007/s10995-015-1908-6 | F |
| 59 | Kinuthia *et al*., 2018 | 10.1186/s12889-018-5567-6 | B |
| 60 | Kirkan *et al*., 2020 | 10.1177/0020764014543713 | D |
| 61 | Koening *et al*., 2006 | 10.2105/AJPH.2005.067744 | F |
| 62 | Kothari *et al*., 2016 | 10.1007/s10995-016-1925-0 | F |
| 63 | Kothari *et al*., 2015 | 10.1002/eat.22429 | E |
| 64 | Leone *et al*., 2010 | 10.1089/jwh.2009.1716 | C |
| 65 | Lukasse *et al*., 2015 | 10.1186/s12884-015-0558-4 | G |
| 66 | Mahengue *et al*., 2016 | 10.1371/journal.pone.0164376 | F |
| 67 | Mahengue *et al*., 2018 | 10.1016/j.jad.2017.12.036 | F |
| 68 | Marks *et al*., 2006 | 10.1016/j.amepre.2006.02.005 | E |
| 69 | Martin *et al*., 2006 | 10.1177/1077801205285106 | E |
| 70 | Martínez-Galiano *et al*., 2017 | 10.1016/j.apnr.2017.02.004 | D |
| 71 | McMahon *et al*., 2011. | [10.1016/j.childyouth.2011.06.001](https://doi.org/10.1016/j.childyouth.2011.06.001) | C |
| 72 | Melville *et al*., 2010 | 10.1097/AOG.0b013e3181f60b0a | F |
| 73 | Miura *et al*., 2017 | [10.3389/fpubh.2017.00081](https://doi.org/10.3389/fpubh.2017.00081) | G |
| 74 | Modiba *et al*., 2011 | 10.1016/j.midw.2010.09.008 | A |
| 75 | Moeini *et al*., 2018 | 10.1016/j.jflm.2016.07.002 | B |
| 76 | Mohammad-Alizadeh-Charandabi *et al*., 2016 | 10.1016/j.jflm.2016.07.002 | B G |
| 77 | Molina *et al*., 2011 | 10.1016/j.whi.2011.01.008 | B |
| 78 | Moraes *et al*., 2002 | 10.1016 / s0020-7292 (02) 00250-3 | E |
| 79 | Mpody *et al*., 2019 | 10.1371/journal.pone.0216 | B |
| 80 | Murray *et al*., 2015 | 10.1186/s12884-015-0662-5 | A E |
| 81 | Nasreem *et al*., 2018 | 10.1186/s12888-018-1781-0 | F |
| 82 | Nelson *et al*., 2018 | 10.24095/hpcdp.38.7/8.01 | F |
| 83 | Njim *et al*., 2018 | 10.1186/s13104-018-3979-0 | D |
| 84 | Ntaganira *et al*., 2009 | 10.1186/1472-6874-8-17 | F |
| 85 | Ogbo *et al*., 2019 | 10.3390/ijerph16162945 | F |
| 86 | Okada *et al*., 2015 | 10.1590/1982- 0194201500045 | A |
| 87 | Oliveira *et al*., 2017 | 10.1007/s00737-016-0705-6 | B |
| 88 | Olofinbiyi *et al*., 2013 | 10.1016/j.ijgo.2013.06.017 | A |
| 89 | Onono *et al*., 2014 | 10.1186/1471-2458-14-390 | F |
| 90 | Peltzer *et al*., 2013 | 10.1007/s10461-012-0185-2 | F |
| 91 | Pereira *et al*., 2009 | 10.1590/s0102-311x2009001200019 | D |
| 92 | Poles *et al*., 2018 | 10.1590/1982- 0194201800050 | A |
| 93 | Pun *et al*., 2018 | 10.1371/journal.pone.0200234 J | A |
| 94 | Quelopana *et al*., 2012 | 10.1080/03630242.2012.687443 | F |
| 95 | Rahman *et al*., 2015 | 10.1363/4108015 | F |
| 96 | Raj *et al*., 2016 | 10.1371/journal.pone.0153190 | B E |
| 97 | Ramezani *et al*., 2015 | [10.22038 / ijp.2015.4858](https://www.researchgate.net/deref/http%3A%2F%2Fdx.doi.org%2F10.22038%2Fijp.2015.4858?_sg%5B0%5D=xZ72Ir1-jD8CcpCKVnRzldOaoOeZ5_RgD-ILgWPP6-fq59k6jRp3RB9G3mFiF6tTrHWyo4b4KfujcCwMXEPb8odnpw.oUgrCRVXa26dlI_YFQrR2ydB5RGclUqwdblRrIWjWIViZJdcNWYJBgAHW8c5y6VNCc_bGJ4qJ-NFk_C26ly-IA) | A |
| 98 | Rishal *et al*., 2018 | 10.1177/1403494817723195 | A |
| 99 | Rodriguez *et al*., 2010 | 10.1177/1077801210366959 | F |
| 100 | Rogathi *et al*., 2017 | 10.1016/j.jad.2017.04.063 | G |
| 101 | Rosen *et al*., 2007 | 10.1177/0886260507304551 | C |
| 102 | Saito *et al*., 2013 | 10.1111/j.1442-2018.2012.00735.x | E |
| 103 | Salazar *et al*., 2012 | 10.1186/1471-2431-12-82 | G |
| 104 | Schneider *et al*., 2018 | 10.1017/gmh.2018.1 | B |
| 105 | Sgobero *et al*., 2015 | 10.5294/aqui.2015.15.3.3 | F |
| 106 | Shrestha *et al*., 2016 | 10.1186/s12884-016-1166-7 | A |
| 107 | Shwartz, 2019 | 10.1016/j.midw.2018.12.011 | C |
| 108 | Sigalla *et al*., 2017 | 10.1371/journal. pone.0172540 | G |
| 109 | Silove *et al*., 2016 | 10.1016/j.jad.2016.07.052 | F |
| 110 | Silverman *et al*., 2016 | 10.1007/s10995-015-1814-y. | F |
| 111 | Spangaro *et al*., 2010 | 10.1080/03630241003705060 | F |
| 112 | Speizer *et al*., 2009 | 10.2105/AJPH.2008.136606) | B F |
| 113 | Sprague *et al*., 2018 | 10.1057/978-1-137-55997-5 | B |
| 114 | Stark *et al*., 2007 | 10.1371/journal. pone.0174741 | F |
| 115 | Steele *et al*., 2019 | [10.1371/journal.pone.0216449](https://doi.org/10.1371/journal.pone.0216449) | F |
| 116 | Stein *et al*., 2015 | 10.1016/j.jneumeth.2015.03.016 | F |
| 117 | Steinberg *et al*., 2008 | 10.1016/j.socscimed.2008.03.033 | F |
| 118 | Stewart *et al*., 1993 | .. | A |
| 119 | Stöckl *et al*., 2010 | 10.1016/j.ijgo.2010.06.014 | A |
| 120 | Thanh Nguyen *et al*., 2016 | 10.1371/journal.pone.0162844 | G |
| 121 | Tho Tran *et al*., 2018 | 10.1371/journal.pone.0207108 | F |
| 122 | Thomas *et al*., 2017 | 10.1016/j.ypmed.2017.07.004 | F |
| 123 | Tiruneh *et al*., 2018 | 10.1080/03630242.2017.1377800 | F |
| 124 | Toan Van *et al*., 2018 | 10.1371/journal.pone.0206650 | G |
| 125 | Tol *et al*., 2018 | 10.1093/ije/dyw130 | F |
| 126 | Trillingsgaard, 2019 | 10.1371/journal. pone.0223824 | E |
| 127 | Turan *et al*., 2016 | 10.1080/17441692.2015.1030684 | E |
| 128 | Urquia *et al*., 2011 | 10.1186/1471-2393-11-42 | C |
| 129 | Valentine *et al*., 2011 | 10.1007/s00737-010-0191-1 | F |
| 130 | Valladares *et al*., 2009 | 10.1080/00016340903015321 | G |
| 131 | Van Parys *et al*., 2015 | 10.1186/s12884-015-0710-1 | G |
| 132 | Vatnar *et al*., 2010 | 10.1177/0886260508329129 | E F |
| 133 | Wangel *et al*., 2016 | 10.1016/j.srhc.2016.04.003 | F |
| 134 | Wikman *et al*., 2019 | 10.1002/jnr.24390 | F |
| 135 | Willie *et al*., 2018 | 10.1080/07399332.2018.1490740 | B F |
| 136 | Wilson *et al*., 2019 | [10.1016/j.midw.2018.10.019](https://doi.org/10.1016/j.midw.2018.10.019) | C |
| 137 | Wojcicki *et al*., 2015 | 10.1371/journal.pone.0118711 | F |
| 138 | Woldetensay *et al*., 2018 | 10.1186/s12884-018-2009-5 | D |
| 139 | Yenes *et al*., 2012 | 10.1007/s10995-011-0747-3 | F |
| 140 | Yohannes *et al*., 2019 | 10.1186/s12978-019-0694-9 | G |
| 141 | Zhong *et al*., 2016 | 10.1016/j.ajog.2016.04.052 | E |

* A: either more than one perpetrator or the perpetrator is not the partner; B: the study sample has been previously selected, i.e.: adolescents, HIV pregnant women, high risk pregnant women; C: study data proceeds from previous surveys carried out for other purposes; D: the study does not provide IPV prevalence; E: either the design used is not appropriated (qualitative studies, case-control studies) or the sample is made up of pregnant victims of IPV; F: the study sample does not include pregnant women, or IPV does not occur during pregnancy or the study does not distinguish between violence produced before and after pregnancy; G: the study sample has been already analysed in another article; H: it does not inform about the country; I: pregnancy is not recent (it happened more than 6 months ago).

**Table S2. Characteristics of the selected studies.**

| **First author** | **Year** | **Country** | **Sample characteristics** | **Time of sample recruitment** | **Time of assessment** | **Period of IPV exposure covered** | **Type of violence** | **IPV tool*** | **Assessor** | **Sample size in reported results** | **Response rate** |
| --- | --- | --- | --- | --- | --- | --- | --- | --- | --- | --- | --- |
| Makayoto, LA | 2013 | Kenya (Kisumu) | All pregnant women attending antenatal care at the Kisumu District Hospital | 2010 | Any trimester | Pregnancy | Ph, S, Ps, any | .. | .. | 300 | .. |
| Velasco, C | 2014 | Spain  (Andalusia) | All inpatient pregnant women delivering during the study period | .. | Immediate postpartum | 12 months prior to delivery | Ph, S, Ps, any | ISA* | Healthcare professional trained in IPV assessment | 779 | 96% |
| Escribá-Agüir, V | 2016 | Spain  (Valencian Region) | Pregnant women who are not illiterate on first ANC visit | 2008 - 2009 | Trimester 1 | 12 months prior to the assessment | Ph, Ps, any | ISA* | Healthcare professional trained in IPV assessment | 1329 | 99.5% |
| Nguyen, TH | 2018 | Vietnam (Dong Anh) | Pregnant women with less than 24 weeks of gestation | 2014 -2015 | Trimester 3 | Pregnancy | Ph, S, Ps,  any | Ad hoc | Technical professional trained in IPV assessment | 1309 | 97.9% |
| Antoniou, E | 2019 | Greece (Athens) | Pregnant women with an average of 32.95 gestation weeks, who isolate ANC in two general public hospitals | 2009 | Trimester 3 | Pregnancy | Ph | AAS | Healthcare professional | 546 | 100% |
| Hedin, LW | 1999 | Sweden | Pregnant women Swedish (or married to Swedish men) visiting three antenatal clinics situated in three different socio-economic areas | 1996 - 1997 | Any trimester | Pregnancy | Ph, S | SVAW | Technical professional | 207 | .. |
| Khaironisak, H | 2017 | Malaysia | All postnatal women admitted to the postnatal ward and mothers who had had abortions and were admitted for a medical procedure, Malaysian citizens and over 18 years | 2015 | Immediate postpartum | Pregnancy | Ph, Ps,  S | WHO | Healthcare professional trained in IPV assessment (women) | 1200 | 99.1% |
| Ludermir, AB | 2014 | Brazil (Pernambuco) | All pregnant women aged 18-49 years, in the third trimester, registered in the Family Health Strategy | 2005 - 2006 | Trimester 3 | Pregnancy | Ps,  any | WHO | Technical professional trained in IPV assessment (women) | 1120 | 98.8% |
| Owaka, IO | 2017 | Kenya  (West Pokot) | Pregnant women attending prenatal clinics | 2014 | Any trimester | Pregnancy | Ph, S, Ps, any | .. | .. | 224 | 94.1% |
| Farid, M | 2008 | Pakistan  (Sindh) | All women between the ages of 15 and 49 years who presented to hospital to deliver, and who had been resident in Karachi for at least 1 year. | 2005 | Trimester 3 | Pregnancy | Ph, Ps, any | WHO | Technical professional trained in IPV assessment (women) | 500 | .. |
| Jamali, S | 2019 | Iran  (Fars) | Iranian women, 15-40 years old in the last month of pregnancy, without addictions | 2017 | Trimester 3 | Pregnancy | Ph, Ps, any | Ad hoc | Healthcare professional trained in IPV assessment (women) | 528 | .. |
| Maciel, MNA | 2019 | France | Puerperal older than 18 years admitted to public and private maternity units throughout France | 2016 | Immediate postpartum | Pregnancy | Ph | .. | .. | 12330 | 88.2% |
| Sigalla, GN | 2017 | Tanzania  (Kilimanjaro) | Pregnant women 18 years of age or older and less than 24 weeks gestation giving birth in Moshi | 2014 - 2015 | Trimester 3 | Pregnancy | Ph, S, Ps, any | WHO | Healthcare professional trained in IPV assessment (women) | 1116 | .. |
| Komori, K | 2019 | Japan | All pregnant women living in 15 areas of Japan (Hokkaido, Miyagi, Fukushima, Chiba, Kanagawa, Koshin, Toyama, Aichi, Kyoto, Osaka, Hyogo, Tottori, Kochi, Fukuoka, and south Kyushu/Okinawa) | 2011 - 2014 | Trimester 2 or 3 | Pregnancy | Ph, Ps | .. | Self-administered | 79985 | 95.82% |
| Shamu, S | 2013 | Zimbabwe (Harare) | Puerperal from 15 to 49 years old who go to 6 primary health care centres | 2011 | 10 days postpartum or 6 weeks postpartum | 12 months prior to the assessment | Ph, S, Ps, any | WHO | Unskilled local staff trained in IPV assessment (women) | 2042 | 97.1% |
| Raffo, JE | 2010 | USA  (Michigan) | Medicaid-insured pregnant women enrolled in a state-wide enhanced prenatal services program | 2005 - 2008 | Any trimester | Pregnancy | Ph, Ps, any | AAS | Healthcare professional | 3675 | .. |
| Gebrezgi, BH | 2017 | Ethiopia  (Tigray) | Pregnant women who attended ANC in public health facilities and who resided in Shire Endaselassie town for at least six months | 2015 | Any trimester | Pregnancy | Ph, any | WHO | Healthcare professional and technical professional trained in IPV assessment | 422 | 100% |
| Varma, D | 2007 | India (Karnataka) | Pregnant women from 18 to 49 years old who come to the ANC clinic and speak English or Kannada | .. | Any trimester | 12 months prior to the assessment | Ph, Ps, S | ISA* | Technical professional trained in IPV assessment | 203 | .. |
| Lau, Y | 2008 | China  (Hong Kong) | All the Chinese women without addictions that  gave birth in the regional public hospital | 2002 - 2003 | Immediate postpartum | Pregnancy | Ps, any | CTS-2* | Technical professional | 1200 | 94.9% |
| Jamshidimanesh, M | 2013 | Iran | Pregnant women entering to give birth | 2010 | .. | Pregnancy | Ph, Ps, any | AAS | .. | 600 | .. |
| Fekadu, E | 2018 | Ethiopia (Amhara) | Pregnant women from 18 to 49 years old who go to ANC to the hospital | 2016 | Any trimester | Pregnancy | Ph, S, Ps, any | WHO | Healthcare professional trained in IPV assessment | 450 | 100% |
| Yu, H | 2018 | China  (Hubei) | Pregnant women attending prenatal exams or hospital deliveries | 2013 - 2014 | Trimester 3 | Pregnancy | Ph, S, Ps, any | AAS | .. | 797 | .. |
| Coonrod, DV | 2007 | USA  (Arizona) | All women 18 years or older presenting for a first prenatal visit | .. | Trimester not specified | Pregnancy | Ph, Ps, S, | CTS* | .. | 342 | 76.6% |
| Gurkan, OC | 2020 | Turkey | Pregnant women presenting to the antenatal polyclinic 18 to 40 years of age, in their 36th to 40th GW of a singleton pregnancy, with no systemic disease and without prenatal risk | 2015 - 2016 | Trimester 3 | Pregnancy | Ph, S, Ps, any | DVAWS | .. | 370 | .. |
| Mammadov, B | 2018 | Cyprus | Pregnant women who were admitted to  the Department of Obstetrics the university hospital | 2015 | Any trimester | Pregnancy | Ph, S, Ps, any | Ad hoc | .. | 219 | 94.8% |
| Mezzavilla, RS | 2016 | ´  Brazil  (Rio de Janeiro) | Mothers who attend primary health care with their children and who have already participated in the first 2 interviews of another cohort. | 2005 - 2009 | 2 months after delivery | 12 months prior to the assessment | Ph | CTS | Healthcare professional trained in IPV assessment | 604 | .. |
| Scrafford, KE | 2019 | USA  (Indiana) | Pregnant women from 18 to 39 years old recruited from a local Women, Infants, and Children office | .. | Any Trimester | 12 months prior to the assessment | Ph, S, Ps, any | CTS-2 | Technical professional trained in IPV assessment | 76 | .. |
| Viellas, EF | 2013 | Brazil  (Rio de Janeiro) | Mothers from 10 to 34 years admitted for childbirth | 2000 - 2001 | Immediate postpartum | Pregnancy | Ph | .. | .. | 8961 | .. |
| Azene, ZN | 2019 | Ethiopia (Amhara) | All pregnant women who come to receive ANC services | 2018 | Any trimester | Pregnancy | Ph, S, Ps, any | WHO | Healthcare professional trained in IPV assessment | 409 | 97.8% |
| Belay, S | 2019 | Ethiopia (Southern Nations, Nationalities and Peoples Region) | Pregnant women living in Wondo-Genet district with gestational age 25–34 weeks | 2017 | Trimester 2 or 3 | Pregnancy | Ph, S, Ps, any | WHO | Technical professional trained in IPV assessment | 589 | 97% |
| Das, S | 2013 | India (Maharashtra) | Postpartum women from 48 slums of Mumbai | 2009 | 6 weeks after delivery | 12 months prior to the assessment | Ph, S, Ps, any | Ad hoc | Technical professional | 2139 | .. |
| Ibrahim, ZM | 2015 | Egypt (Ismailia) | All pregnant women aged 18 to 43 attending the Obstetrics outpatient clinic of University Hospital | 2010 - 2012 | Any trimester | Pregnancy | Ph, S, Ps, any | NorAQ | .. | 1857 | .. |
| Lukasse, M | 2014 | Belgium | Pregnant women over 18 years cared for in ANC services | 2008 - 2010 | Any trimester | 12 months prior to the assessment | Ph, S, Ps, any | NorAQ | Self-administered | 861 | 50 – 78% |
| Lukasse, M | 2014 | Iceland | Pregnant women over 18 years cared for in ANC services | 2008 - 2010 | Any trimester | 12 months prior to the assessment | Ph, S, Ps, any | NorAQ | Self-administered | 602 | 57.3 – 65% |
| Lukasse, M | 2014 | Denmark | Pregnant women over 18 years cared for in ANC services | 2008 - 2010 | Any trimester | 12 months prior to the assessment | Ph, S, Ps, any | NorAQ | Self-administered | 1290 | 57.3 – 65% |
| Lukasse, M | 2014 | Estonia | Pregnant women seen at ANC services without major fetal pathologies | 2008 - 2010 | Any trimester | 12 months prior to the assessment | Ph, S, Ps, any | NorAQ | Self-administered | 975 | 90% |
| Lukasse, M | 2014 | Norway | Pregnant women seen at ANC services without major fetal pathologies | 2008 - 2010 | Any trimester | 12 months prior to the assessment | Ph, S, Ps, any | NorAQ | Self-administered | 2424 | 50% |
| Lukasse, M | 2014 | Sweden | Pregnant women seen at ANC services without major fetal pathologies | 2008 - 2010 | Any trimester | 12 months prior to the assessment | Ph, S, Ps, any | NorAQ | Self-administered | 1022 | 50 – 78% |
| Nasreen, HE | 2011 | Bangladesh (Mymensingh) | Pregnant women residing in the area and in the third trimester | 2008 - 2009 | Trimester 3 | Pregnancy | Ph | .. | Technical professional trained in IPV assessment  (women) | 720 | .. |
| Oweis, A | 2010 | Jordan  (Irbid) | Pregnant women who go to 5 public health centres | 2006 | Any trimester | Pregnancy | Ph, S, Ps | .. | Self-administered | 316 | 87.4% |
| Abebe Abate, B | 2016 | Ethiopia (Oromia) | Pregnant women aged 15–49 years who are living in the study area for at least six months | 2014 | Any trimester | .. | Ph, S, Ps, any | WHO | Technical professional trained in IPV assessment  (women) | 282 | 94.3% |
| Alhusen, JL | 2013 | USA (Maryland) | Pregnant women over 16 years, in WG 24-28 and without chronic pathology, attended in 3 obstetric clinics | 2009 - 2010 | Trimester 2 | Pregnancy | Ph | AAS | Technical professional | 166 | .. |
| Chan, KL | 2009 | China  (Hong Kong) | Pregnant women from 18 to 50 years old, in WG 36, who go to the obstetrics unit of 7 hospitals | 2005 -2006 | Trimester 3 | Pregnancy | Ph, S, Ps | AAS | Healthcare professional trained in IPV assessment  (women) | 3245 | 93.7% |
| Ezeudu, CC | 2019 | Nigeria (Enugu) | Mothers who go to postnatal care services to immunize their children | 2015 | 3 months after delivery | Pregnancy | Ph, Ps, any | .. | Technical professional trained in IPV assessment | 702 | .. |
| Johri, M | 2011 | Guatemala | Pregnant women reporting to the maternity ward of the hospital general | 2006 | Any trimester | 12 months prior to the assessment | Ph, S, Ps, any | Ad hoc | Technical professional | 1897 | 91.6% |
| Fonseca-Machado, MO | 2015 | Brazil  (Sao Paulo) | Pregnant women between 15 and 49 years of age attending the prenatal outpatient clinic in the third trimester | 2012 - 2013 | Trimester 3 | Pregnancy | Ph, S, Ps, any | WHO | Technical professional trained in IPV assessment | 358 | .. |
| Lencha, B | 2019 | Ethiopia (Oromia) | Pregnant women who were attending ANC clinic in hospital | .. | Any trimester | Pregnancy | Ph, S, Ps, any | WHO | Technical professional trained in IPV assessment (women) | 612 | 97.8% |
| Mahenge, B | 2013 | Tanzania  (Dar es Salaam) | Pregnant women attending the antenatal care clinic of National Hospital | 2011 - 2012 | Any trimester | Pregnancy | Ph , S | CTS | Healthcare professional trained in IPV assessment | 1180 | .. |
| Ogunwale, AN | 2017 | USA  (Texas) | Hispanic pregnant women who delivered at a Houston hospital | 2011 - 2012 | Immediate postpartum | Pregnancy | Any | Ad hoc | Self-administered | 613 | 90% |
| Okour, AM | 2011 | Jordan  (Al-Mafraq) | Pregnant women who attended antenatal clinics | 2007 |  | Pregnancy | Ph, S, Ps, any | WHO | Healthcare professional (women) | 303 | 96.8% |
| Perales, MT | 2009 | Perú  (Lima) | Postpartum women aged 15-49 years admitted to hospital | 2005 - 2006 | Immediate postpartum | Pregnancy | Ph, S, Ps, any | Ad hoc | Technical professional trained in IPV assessment | 2392 | 99% |
| Romero-Gutiérrez, G | 2011 | Mexico (Guanajuato) | Postpartum women admitted to a public hospital for childbirth | 2004 - 2006 | Immediate postpartum | Pregnancy | Ph, S, Ps, any | Castro | .. | 1263 | 98.6% |
| Shaikh, MA | 2008 | Pakistan (Punjab) | Pregnant women over 20 visiting public sector hospitals in Islamabad and Rawalpindi | 2006 | .. | Pregnancy | Ph | AAS | Technical professional trained in IPV assessment (women) | 493 | 70.2% |
| Singh, JK | 2018 | Nepal (Janakpur) | Pregnant women from 15 to 45 years old in the second trimester of pregnancy | 2015 | Trimester 2 | .. | Any | National Demographic and Health Survey 2011 in Nepal | Unskilled local staff (women) | 426 | 94.3% |
| Valladares, E | 2005 | Nicaragua (Leon) | Pregnant women from the municipality of Leon | .. | Any trimester | Pregnancy | Ph, S, Ps, any | WHO | Technical professional trained in IPV assessment (women) | 478 | .. |
| Van Parys, AS | 2014 | Belgium (Flanders) | Pregnant women over 18 years attended in hospital prenatal care clinics | 2010 - 2012 | Any trimester | Pregnancy |  | AAS* | Self-administered | 1894 | 76.7% |
| Yang, MS | 2006 | Taiwan | Aboriginal women who had just given birth in hospitals | 2003 | Immediate postpartum | Pregnancy | Ph | Ad hoc | .. | 1143 | .. |
| Zhu, Y | 2016 | China  (Anhui) | Pregnant women in their third trimester of pregnancy who were attending primary health-care clinics | 2013 - 2014 | Trimester 3 | Pregnancy | Ph, Ps, S | .. | .. | 928 | .. |
| Chaquisse, E | 2018 | Mozambique (Nampula) | Pregnant women, who visited the primary health care facilities for their first prenatal appointment | 2013 - 2014 | Any trimester | 12 months prior to the assessment | Ph, S | CTS-2 | Healthcare professional trained in IPV assessment (women) | 869 | .. |
| Farrokh-Eslamlou, H | 2014 | Iran  (West Azerbaijan) | Postpartum women residing in Urmia who go to health centres for their child's first vaccination | 2012 | Immediate postpartum | Pregnancy | Ph, S, Ps, any | AAS | Healthcare professional trained in IPV assessment | 313 | 89.4% |
| Ferdos, J | 2018 | Bangladesh (Rajshahi) | Postpartum women admitted to postpartum rooms | 2015 - 2016 | Immediate postpartum | Pregnancy | Ph, S | CTS | Technical professional trained in IPV assessment (women) | 400 | .. |
| Jain, S | 2017 | India  (Delhi) | Pregnant women from 20 to 28 WG who come to ANC in the hospital | 2013 - 2015 | Trimester 2 | Pregnancy | Ph, S, Ps, any | Ad hoc | .. | 400 | .. |
| Jackson, CL | 2015 | USA  (Arizona) | Low-income Mexican American pregnant women over 18 years of age, attended at two prenatal clinics in the public sector | .. | Trimester 3 | Pregnancy | Ph, S, Ps,  any | 14 items from the Pregnancy Risk Assessment Monitoring System | Technical professional trained in IPV assessment (women) | 320 | .. |
| Kataoka, Y | 2016 | Japan  (Nagano) | Pregnant women recruited at two prenatal clinics in the general hospital | 2011 - 2012 | Trimester 3 | Pregnancy | Ph, S,  any | VAWS | Self-administered | 84 | 89.5% |
| Kita, S | 2014 | Japan  (Tokio) | Pregnant women with more than 10 weeks of gestation and without serious mental illness who visit a maternity clinic | 2009 | Any trimester | Pregnancy | Ph, S, Ps,  any | VAWS | Healthcare professional trained in IPV assessment  (women) | 302 | 100% |
| Marcacine, KO | 2013 | Brazil  (Sao Paulo) | Mothers attending the postpartum review | 2011 - 2012 | 45 -60 days postpartum | Pregnancy | Ph, S, Ps,  any | Ad hoc | Healthcare professional trained in IPV assessment | 207 | .. |
| Martin, KR | 2011 | USA (California) | Latino pregnant women attending five obstetric clinics serving low-income populations | 1998 - 2000 | Trimester 2 or 3 | Pregnancy | Ph, Ps | Castro | Technical professional trained in IPV assessment (women) | 313 | .. |
| Moghaddam Hossieni, V | 2017 | Iran  (Razavi Khorasan) | Pregnant women between 18 and 40 years of Iranian nationality and Farsi-speaking, with a single low-risk pregnancy and more than 14 WG, who go to the health centre | .. | Trimester 2 or 3 | 12 months prior to the assessment | Ph, S, Ps,  any | CTS-2 | Self-administered | 174 | 82.8% |
| Rodrigues, DP | 2014 | Brazil  (Sao Paulo) | Pregnant women between 15 and 49 years of age receiving health care in a public hospital | 2012 | Any trimester | Pregnancy | Ph, S, Ps,  any | .. | Technical professional trained in IPV assessment | 232 | .. |
| Roelens, K | 2008 | Belgium (Flanders) | Dutch-speaking pregnant women attending five large hospitals in the province of East Flanders | 2003 | Any trimester | Pregnancy | Ph, S | AAS | Self-administered | 537 | 39.4% |
| Rurangirwa, AA | 2017 | Rwanda (Kigali) | Women who gave birth within the past 13 months | 2014 | 0-13 months postpartum | Pregnancy | Ph, S, Ps | WHO | Healthcare professional trained in IPV assessment | 922 | .. |
| Thakur, A | 2018 | India  (Madhya Pradesh) | Women admitted to the postpartum ward of nine public hospitals in Madhya Pradesh | 2016 | Immediate postpartum | Pregnancy | Ph, S, Ps,  any | Ad hoc | Healthcare professional trained in IPV assessment | 3839 | 98.5% |
| Amemiya, A | 2016 | Japan  (Aichi) | Mothers who enrol in a health monitoring program for their 4-month-old children | 2012 | 4 months after delivery | Pregnancy | Ph, Ps | .. | .. | 6590 | 68% |
| Anderson, BA | 2017 | USA (California) | Hispanic and non-Hispanic white pregnant women living under severe economic and cultural tensions who attend a prenatal visit | 2000 | Trimester not specified | .. | Any | AAS | Healthcare professional trained in IPV assessment | 109 | .. |
| Ayodapo, OA | 2017 | Nigeria  (Oyo) | Pregnant women between the ages of 18 and 49 who attended for antenatal visits | 2010 | Any trimester | Pregnancy | Ph, S, Ps | .. | .. | 350 | 88.6% |
| Bolu, F | 2015 | Turkey (Düzce) | Pregnant women attending a prenatal clinic | 2012 - 2013 | Trimester not specified | Pregnancy | Ph, S, Ps, any | .. | Self-administered | 191 | .. |
| Connelly, CD | 2013 | USA (California) | Women receiving obstetric healthcare during pregnancy and up to 6 months postpartum | 2009 - 2012 | Any trimester and postpartum (up to 6 months) | 12 months prior to the assessment | Any | AAS | Technical professional | 1868 | 86.8% |
| Costa-Ribeiro, MR | 2014 | Brazil (Maranhao) | Women with simple pregnancy, ultrasound examination before 20 WG, who come to prenatal control of three public hospitals and who are going to give birth in Saint Louis | 2010 - 2011 | Trimester 2 | Pregnancy | Ps | WHO | .. | 982 | .. |
| Finnbogadottir, H | 2014 | Sweden (Scania) | Women ≥ 18 years, registered at antenatal care when pregnant and who understand and write Swedish or English | 2012 - 2013 | Trimester 2 or 3 | Pregnancy | Ph, Ps, S | NorAQ | Self-administered | 1939 | .. |
| Gashaw, BT | 2018 | Ethiopia  (Oromia) | Pregnant women with more than 24 WG who come to ANC | 2015 - 2016 | Trimester 2 and 3 | Pregnancy | Any | Ad hoc | Healthcare professional trained in IPV assessment (women) | 720 | .. |
| Henriksen, L | 2013 | Norway | Norwegian resident pregnant women | 1999 - 2008 | Trimester 2 | 12 months prior to the assessment | S | NorAQ | .. | 78660 | 38.7% |
| Iliyasu, Z | 2013 | Nigeria (Kano) | Pregnant women attending ANC at Hospital | .. | Any trimester | Pregnancy | Ph, S, Ps,  any | CTS-2 | Technical professional trained in IPV assessment (women) | 392 | 98% |
| Jamali, S | 2017 | Iran (Fars) | Pregnant women without mental disorder or addictions receiving routine prenatal care | 2016 | Any trimester | Pregnancy | Ph, S, Ps,  any | .. | .. | 1097 | 84.4% |
| Kita, S | 2016 | Japan  (Tokio) | Pregnant women with more than 28 WG, without serious mental illness attending ANC | 2013 - 2014 | Trimester 3 | Pregnancy | Ps | ISA | Self-administered | 562 | 87.1% |
| Phaswana-Mafuya, N | 2009 | South Africa (Mpumalanga) | Pregnant women attending public prenatal health services at 35 Gert Sibande district clinics | .. | Any trimester | 12 months prior to the assessment | Ph, Ps, S | CTS-2 | Technical professional | 984 | .. |
| Salazar, M | 2009 | Nicaragua (Leon) | Pregnant women selected from León Health and Demographic Surveillance System baseline | 2002 - 2003 | Postpartum | Pregnancy | Any | WHO | Technical professional trained in IPV assessment (women) | 398 | .. |
| Shamu, S | 2016 | Zimbabwe (Harare) | Puerperal women from 15 to 49 years old who attend postnatal clinics | 2011 | Postpartum | Pregnancy | Ph, S, Ps,  any | WHO | Technical professional trained in IPV assessment (women) | 842 | .. |
| Valencia, ES | 2010 | Brazil  (Rio de Janeiro) | Third-trimester pregnant women attending a public health centre | 2006 - 2007 | Trimester 3 | 12 months prior to the assessment | Ph, S | AAS | Healthcare professional trained in IPV assessment | 331 | .. |
| Yanikkerem, E | 2006 | Turkey (Manisa) | All pregnant women living in the selected health areas (one rural and one urban) | 2004 | Any trimester | Pregnancy | Ph, Ps, S and any | DVAWS | .. | 217 | 88.2% |
| Zhang, Y | 2012 | China  (Hunan) | All women who had a normal spontaneous or caesarean delivery at the Women and Child Health Centre in Changsha | 2006 -2007 | Immediate postpartum | Pregnancy | Ps, any | AAS | Healthcare professional trained in IPV assessment | 846 | .. |
| Abdollahi, F | 2015 | Iran (Mazandaran) | All pregnant women between 18 and 45 years of age who attend prenatal care in urban primary health care centres in the first trimester of pregnancy | 2010 | Trimester 1 | Pregnancy | Ph | WHO | Technical professional trained in IPV assessment | 1461 | 94.3% |
| Ferraro, AA | 2017 | Brazil  (Sao Paulo) | All pregnant women who were attending pre-natal care in 5 primary care facilities in the region of Butantan | 2010 - 2012 | Trimester 3 | 12 months prior to the assessment | Ph, S, Ps,  any | WHO | Healthcare professional trained in IPV assessment | 900 | .. |
| Malan, M | 2018 | South Africa (Western Cape) | Pregnant women over 18 years of age attending prenatal care in units of midwife and obstetrics | 2015 | Any trimester | 12 months prior to the assessment | Ph, S, Ps,  any | WHO | Healthcare professional | 150 | .. |
| Islam, MDJ | 2017 | Bangladesh (Chittagong) | Married women aged between 15 and 49 years, living with their husbands for minimum last 2 years, and who were in the first 6 months postpartum | 2015 - 2016 | Postpartum | Pregnancy | Ph, S, Ps,  any | WHO | Unskilled local staff | 426 | .. |
| Bailey, BA | 2007 | USA (Tennessee) | Pregnant women attending a prenatal visit in a family medicine centre | .. | Trimester 3 | 12 months prior to the assessment | Ph, S, Ps,  any | CTS-2 | Self-administered | 104 | 97% |
| Onoh, RC | 2013 | Nigeria (Ebonyi) | Married pregnant women attending prenatal care at least a second time | 2011 | Trimester 2 or 3 | Pregnancy | S, Ps,  any | .. | Self-administered | 321 | 91.7% |
| Boonnate, N | 2015 | Thailand (Chiang Mai) | Pregnant women over 18 years of age, in their week of gestation 32-40 and without psychiatric pathology in treatment, who attend routine prenatal control | .. | Any trimester | Pregnancy | Any | ISA | .. | 230 | .. |
| Chan, YY | 2019 | Malaysia  (all the states) | Women at 6 to 16 weeks postpartum who attended the selected government primary health care clinics | 2016 | Postpartum | 12 months prior to the assessment | Ph, S, Ps,  any | WHO | Healthcare professional trained in IPV assessment (women) | 5727 | 85.9% |
| Altarac, M | 2002 | USA  (Maryland) | Pregnant women older than 18 years and more than 20 weeks' gestation who gave birth at JHH between February 1995 and May 1996, and who have had one or no prenatal care visits or who have received prenatal care through 4 partner clinics with the Johns Hopkins Health System | 1995-1996 | Immediate postpartum | Pregnancy | Ph | CTS | .. | 808 | 88.7% |
| Gibbs, A | 2017 | South Africa (KwaZulu-Nata) | Mothers over 18 years of age, babies under 6 weeks, who go to a public health centre | 2015 | Postpartum | 12 months prior to the assessment | Any | WHO | Technical professional (women) | 275 | 91.9% |
| Silva, EP | 2011 | Brazil (Pernambuco) | All pregnant women between the ages of 18 and 49 enrolled in the Family Health Program | 2005 - 2006 | Any trimester | Pregnancy | Ph, S, Ps,  any | WHO | Technical professional trained in IPV assessment | 960 | 98.9% |
| Deveci, SE | 2007 | Turkey  (Elazig) | Pregnant women who attended antenatal clinics | .. | Trimester not specified | Pregnancy | Ph, Ps, S | Ad hoc | Healthcare professional trained in IPV assessment | 249 | .. |
| Gómez-Beloz, A | 2008 | Peru  (Lima) | Women who delivered at the Instituto Nacional Materno Perinatal (INMP) in Lima | 2005 - 2006 | Immediate postpartum | Pregnancy | Any | Ad hoc | Technical professional trained in IPV assessment | 2317 | 99.9% |
| Karmaliani, R | 2008 | Pakistan (Sindh) | Pregnant women from 20 to 26 weeks of gestation who come to prenatal control and who will reside in the city in the next 6 months | .. | Trimester 2 or 3 | Pregnancy | Any | Ad hoc | Healthcare professional trained in IPV assessment (women) | 1324 | 94% |
| Bhatta, N | 2019 | Nepal (kathmandu) | Healthy pregnant or postpartum women aged 15-49 years attending the referral hospital in Kathmandu, either for antenatal care or for immunization of their children | 2017 - 2018 | Any trimester | Pregnancy | Ph, Ps, S, any | WHO | Healthcare professional trained in IPV assessment | 495 | 100% |
| Idoko, P | 2015 | Gambia (Banjul) | Pregnant women in the first prenatal care visit | 2012 | Trimester not specified | Pregnancy | Ph, Ps, S, any | Ad hoc | Healthcare professional | 136 | 84.5% |
| Adebowale, O | 2020 | Nigeria  (Edo) | Pregnant women aged 16-44 receiving prenatal care at a secondary level health center in southern Nigeria | 2015-2016 | Trimester not specified | 12 months prior to the assessment | Ph, Ps, any | CAS | .. | 395 | 98.7% |
| Akaba, GO | 2020 | Nigeria (Abuja) | All the women who gave birth at the University Hospital of Abuja | 2017 | Immediate postpartum | 12 months prior to the assessment | Any | Any | .. | 349 | 94.8% |
| Kana, MA | 2020 | Nigeria (Kaduna) | Postpartum women after 14 weeks or less after delivery, who come to vaccinate their newborn | 2018 | Postpartum | Pregnancy | Ph, Ps, S, any | CTS | .. | 293 | .. |
| Luhumyo, L | 2020 | Kenya  ([Uasin Gishu](https://es.wikipedia.org/wiki/Condado_de_Uasin_Gishu)) | All puerperas of the Moi Teaching and Referral Hospital | 2017 | Immediate postpartum | Pregnancy | Ph, Ps, S, any | WHO | Healthcare professional and technical professional trained in IPV assessment | 369 | 96.8% |
| McKelvie, S | 2020 | Republic of Vanuatu  (Sanma) | All pregnant women over 18 who receive prenatal care at the Northern Provincial Hospital (NPH) in the municipality of Luganville | 2019 | Any trimester | Pregnancy | Ph, Ps, S, any | WHO | Technical professional trained in IPV assessment | 187 | .. |
| Navarrete, L | 2020 | Mexico  (Mexico City) | Pregnant women older than 20 years and more than 26 weeks of gestation, living in the metropolitan area of Mexico and attending prenatal consultation | .. | Trimester 2 or 3 | Pregnancy | Any | Ad hoc | Healthcare professional trained in IPV assessment | 280 | 62.9% |
| Silva, RP | 2020 | Brasil (Cariacica) | Puerperal women hospitalized with at least 24 hours after childbirth and live fetus (>500 grams) who had an intimate partner during pregnancy | 2017 | Immediate postpartum | Pregnancy | Ph, Ps, S | WHO | Technical professional trained in IPV assessment  (women) | 330 | .. |

Ph, Physical violence; Ps, psychological violence; S, sexual violence; Any, any kind of violence; ISA, Index of Spouse Abuse; AAS, Abuse assessment screen; SVAWS, Severity of Violence Against Women Scale; WHO, World Health Organization; CTS, Conflict Tactics Scale; DVAWS, Domestic Violence Against Women Screening Form; SVAWS, NorVold Abuse Questionnaire; VAWS, Canada Violence Against Women Survey; CAS, Composite Abuse Scale

.. No data

* The author used more than one tool

**Table S3. Intimate partner violence during pregnancy by country and IPV type**

| **IPV type** | **Country** | **Studies** | **N** | **n** | **Effect (95% CI)** |
| --- | --- | --- | --- | --- | --- |
| **Physical** |  |  |  |  |  |
|  | Belgium | 3 | 3292 | 40 | 0.01 (0.00, 0.02) |
|  | Slovenia | 1 | 1018 | 10 | 0.01 (0.00, 0.02) |
|  | Cyprus | 1 | 219 | 2 | 0.01 (0.00, 0.02) |
|  | Japan | 4 | 86961 | 1062 | 0.01 (0.01, 0.01) |
|  | Malaysia | 2 | 6927 | 203 | 0.01 (0.01, 0.01) |
|  | United Kingdom | 1 | 7591 | 78 | 0.01 (0.01, 0.01) |
|  | Canada | 2 | 4850 | 68 | 0.01 (0.01, 0.02) |
|  | Spain | 2 | 2098 | 86 | 0.01 (0.01, 0.02) |
|  | Iceland | 1 | 602 | 13 | 0.02 (0.01, 0.03) |
|  | France | 1 | 12330 | 220 | 0.02 (0.02, 0.02) |
|  | Denmark | 1 | 1290 | 32 | 0.02 (0.02, 0.03) |
|  | Norway | 1 | 2424 | 55 | 0.02 (0.02, 0.03) |
|  | Switzerland | 1 | 206 | 6 | 0.03 (0.01, 0.05) |
|  | Estonia | 1 | 975 | 27 | 0.03 (0.02, 0.04) |
|  | Vietnam | 1 | 1309 | 46 | 0.04 (0.03, 0.05) |
|  | China | 3 | 2538 | 119 | 0.05 (0.02, 0.07) |
|  | Sweden | 3 | 3168 | 71 | 0.05 (0.02, 0.08) |
|  | Greece | 1 | 546 | 33 | 0.06 (0.04, 0.08) |
|  | Taiwan | 1 | 1143 | 79 | 0.07 (0.05, 0.08) |
|  | Mexico | 3 | 3402 | 270 | 0.09 (0.06, 0.12) |
|  | Guatemala | 1 | 1897 | 181 | 0.10 (0.08, 0.11) |
|  | Rwanda | 1 | 922 | 94 | 0.10 (0.08, 0.12) |
|  | India | 4 | 6581 | 650 | 0.11 (0.08, 0.13) |
|  | Nepal | 1 | 495 | 54 | 0.11 (0.08, 0.14) |
|  | United Republic of Tanzania | 3 | 2796 | 341 | 0.12 (0.05, 0.20) |
|  | Brazil | 8 | 12553 | 805 | 0.12 (0.06, 0.17) |
|  | United States of America | 9 | 6354 | 470 | 0.12 (0.08, 0.15) |
|  | Peru | 1 | 2392 | 285 | 0.12 (0.11, 0.13) |
|  | Turkey | 7 | 2212 | 226 | 0.13 (0.07, 0.20) |
|  | Nicaragua | 1 | 478 | 64 | 0.13 (0.10, 0.16) |
|  | Malawi | 1 | 292 | 40 | 0.14 (0.10, 0.18) |
|  | Pakistan | 6 | 2113 | 301 | 0.15 (0.09, 0.20) |
|  | Iran | 7 | 7323 | 1028 | 0.15 (0.10, 0.21) |
|  | South Africa | 3 | 2636 | 305 | 0.16 (0.09, 0.23) |
|  | Ethiopia | 14 | 8859 | 1336 | 0.16 (0.12, 0.20) |
|  | Egypt | 1 | 1857 | 297 | 0.16 (0.14, 0.18) |
|  | Zimbabwe | 2 | 2884 | 477 | 0.16 (0.15, 0.18) |
|  | Solomon Islands | 1 | 242 | 41 | 0.17 (0.12, 0.22) |
|  | Jordan | 2 | 619 | 138 | 0.17 (0.14, 0.20) |
|  | Kenya | 3 | 893 | 151 | 0.18 (0.08, 0.27) |
|  | Nigeria | 6 | 2210 | 387 | 0.19 (0.11, 0.27) |
|  | Uganda | 2 | 792 | 189 | 0.21 (0.18, 0.24) |
|  | Saudi Arabia | 1 | 7105 | 1492 | 0.21 (0.20, 0.22) |
|  | Vanuatu | 1 | 187 | 41 | 0.22 (0.16, 0.28) |
|  | Bangladesh | 3 | 1546 | 436 | 0.31 (0.17, 0.45) |
|  | Mozambique | 1 | 869 | 377 | 0.43 (0.40, 0.47) |
|  | Gambia | 1 | 136 | 75 | 0.55 (0.47, 0.64) |
| **Psychological** |  |  |  |  |  |
|  | Sweden | 2 | 2961 | 29 | 0.01 (0.00, 0.01) |
|  | Canada | 2 | 4850 | 137 | 0.02 (0.01, 0.02) |
|  | Denmark | 1 | 1290 | 28 | 0.02 (0.01, 0.03) |
|  | Iceland | 1 | 602 | 11 | 0.02 (0.01, 0.03) |
|  | Malaysia | 2 | 6927 | 464 | 0.02 (0.02, 0.03) |
|  | Norway | 1 | 2424 | 57 | 0.02 (0.02, 0.03) |
|  | Slovenia | 1 | 1018 | 33 | 0.03 (0.02, 0.04) |
|  | Thailand | 1 | 230 | 10 | 0.04 (0.02, 0.07) |
|  | Spain | 2 | 2099 | 203 | 0.04 (0.03, 0.05) |
|  | Switzerland | 1 | 206 | 10 | 0.05 (0.02, 0.08) |
|  | United Kingdom | 1 | 7591 | 367 | 0.05 (0.04, 0.05) |
|  | Estonia | 1 | 975 | 49 | 0.05 (0.04, 0.06) |
|  | Belgium | 2 | 2755 | 198 | 0.05 (0.05, 0.06) |
|  | China | 6 | 7829 | 775 | 0.11 (0.07, 0.16) |
|  | South Africa | 3 | 2636 | 208 | 0.12 (0.07, 0.18) |
|  | India | 4 | 6581 | 983 | 0.13 (0.06, 0.20) |
|  | Cyprus | 1 | 219 | 28 | 0.13 (0.08, 0.17) |
|  | Japan | 4 | 87439 | 11604 | 0.13 (0.09, 0.17) |
|  | Malta | 1 | 300 | 44 | 0.15 (0.11, 0.19) |
|  | Nepal | 1 | 495 | 74 | 0.15 (0.12, 0.18) |
|  | Peru | 1 | 2392 | 373 | 0.16 (0.14, 0.17) |
|  | Guatemala | 1 | 1897 | 310 | 0.16 (0.15, 0.18) |
|  | Rwanda | 1 | 922 | 157 | 0.17 (0.15, 0.19) |
|  | Brazil | 8 | 5089 | 1308 | 0.24 (0.17, 0.31) |
|  | Uganda | 2 | 792 | 192 | 0.24 (0.21, 0.27) |
|  | Ethiopia | 12 | 8221 | 2004 | 0.25 (0.15, 0.34) |
|  | United Republic of Tanzania | 2 | 1616 | 409 | 0.25 (0.23, 0.27) |
|  | Jordan | 2 | 619 | 159 | 0.26 (0.22, 0.29) |
|  | Malawi | 1 | 292 | 82 | 0.28 (0.23, 0.33) |
|  | Portugal | 1 | 852 | 240 | 0.28 (0.25, 0.31) |
|  | Mexico | 2 | 2091 | 649 | 0.31 (0.29, 0.33) |
|  | Nicaragua | 1 | 478 | 155 | 0.32 (0.28, 0.37) |
|  | Vietnam | 1 | 1309 | 421 | 0.32 (0.30, 0.35) |
|  | Pakistan | 4 | 1360 | 476 | 0.33 (0.08, 0.59) |
|  | Turkey | 7 | 2421 | 654 | 0.33 (0.17, 0.49) |
|  | Egypt | 1 | 1857 | 606 | 0.33 (0.31, 0.35) |
|  | Vanuatu | 1 | 187 | 63 | 0.34 (0.27, 0.40) |
|  | Nigeria | 7 | 2535 | 761 | 0.35 (0.17, 0.54) |
|  | Kenya | 3 | 893 | 313 | 0.37 (0.21, 0.53) |
|  | United States of America | 6 | 4830 | 703 | 0.39 (0.21, 0.56) |
|  | Iran | 6 | 5862 | 2337 | 0.45 (0.26, 0.63) |
|  | Solomon Islands | 1 | 242 | 109 | 0.45 (0.39, 0.51) |
|  | Zimbabwe | 2 | 2884 | 1294 | 0.45 (0.43, 0.47) |
|  | Gambia | 1 | 136 | 82 | 0.60 (0.52, 0.69) |
|  | Bangladesh | 1 | 426 | 277 | 0.65 (0.60, 0.70) |
| **Sexual** |  |  |  |  |  |
|  | Sweden | 4 | 81828 | 276 | 0.00 (0.00, 0.00) |
|  | Norway | 1 | 2424 | 12 | 0.00 (0.00, 0.01) |
|  | Denmark | 1 | 602 | 3 | 0.00 (-0.00, 0.01) |
|  | Iceland | 1 | 975 | 3 | 0.00 (-0.00, 0.01) |
|  | Pakistan | 2 | 560 | 4 | 0.00 (-0.00, 0.01) |
|  | Belgium | 2 | 3292 | 13 | 0.01 (0.00, 0.01) |
|  | Estonia | 1 | 1290 | 8 | 0.01 (0.00, 0.01) |
|  | Japan | 2 | 386 | 7 | 0.01 (0.00, 0.02) |
|  | Malaysia | 2 | 6927 | 154 | 0.01 (0.01, 0.01) |
|  | China | 3 | 2538 | 42 | 0.02 (0.00, 0.03) |
|  | Switzerland | 1 | 206 | 4 | 0.02 (0.00, 0.04) |
|  | Brazil | 8 | 4629 | 116 | 0.02 (0.01, 0.03) |
|  | Guatemala | 1 | 1897 | 61 | 0.03 (0.02, 0.04) |
|  | Spain | 1 | 759 | 23 | 0.03 (0.02, 0.04) |
|  | Uganda | 2 | 792 | 35 | 0.03 (0.02, 0.05) |
|  | South Africa | 3 | 2636 | 68 | 0.04 (0.01, 0.07) |
|  | Peru | 1 | 2392 | 93 | 0.04 (0.03, 0.05) |
|  | India | 4 | 6581 | 564 | 0.06 (0.00, 0.13) |
|  | Mexico | 2 | 2091 | 135 | 0.06 (0.05, 0.07) |
|  | Nicaragua | 1 | 478 | 32 | 0.07 (0.04, 0.09) |
|  | Cyprus | 1 | 219 | 16 | 0.07 (0.04, 0.11) |
|  | Jordan | 2 | 619 | 65 | 0.08 (0.06, 0.11) |
|  | United States of America | 4 | 842 | 62 | 0.10 (0.04, 0.17) |
|  | Rwanda | 1 | 922 | 89 | 0.10 (0.08, 0.12) |
|  | Vietnam | 1 | 1309 | 130 | 0.10 (0.08, 0.12) |
|  | Egypt | 1 | 1857 | 186 | 0.10 (0.09, 0.11) |
|  | Canada | 1 | 100 | 12 | 0.12 (0.06, 0.18) |
|  | Vanuatu | 1 | 187 | 23 | 0.12 (0.08, 0.17) |
|  | Ethiopia | 12 | 8221 | 1063 | 0.14 (0.10, 0.18) |
|  | United Republic of Tanzania | 3 | 2796 | 450 | 0.15 (0.09, 0.21) |
|  | Turkey | 5 | 1556 | 259 | 0.16 (0.07, 0.26) |
|  | Nigeria | 5 | 1442 | 216 | 0.18 (0.11, 0.25) |
|  | Nepal | 1 | 495 | 94 | 0.19 (0.16, 0.22) |
|  | Kenya | 3 | 893 | 172 | 0.21 (0.08, 0.34) |
|  | Gambia | 1 | 136 | 30 | 0.22 (0.15, 0.29) |
|  | Iran | 5 | 5334 | 1187 | 0.22 (0.17, 0.27) |
|  | Bangladesh | 2 | 826 | 184 | 0.22 (0.19, 0.25) |
|  | Malawi | 1 | 292 | 84 | 0.29 (0.24, 0.34) |
|  | Solomon Islands | 1 | 242 | 85 | 0.35 (0.29, 0.41) |
|  | Zimbabwe | 2 | 2884 | 1129 | 0.39 (0.37, 0.41) |
|  | Mozambique | 1 | 869 | 396 | 0.46 (0.42, 0.49) |
| **Any*** |  |  |  |  |  |
|  | Germany | 1 | 401 | 9 | 0.02 (0.01, 0.04) |
|  | Canada | 2 | 4850 | 158 | 0.02 (0.02, 0.02) |
|  | France | 1 | 12330 | 220 | 0.02 (0.02, 0.02) |
|  | Denmark | 1 | 1290 | 42 | 0.03 (0.02, 0.04) |
|  | Slovenia | 1 | 1018 | 33 | 0.03 (0.02, 0.04) |
|  | Sweden | 1 | 1022 | 31 | 0.03 (0.02, 0.04) |
|  | Iceland | 1 | 602 | 20 | 0.03 (0.02, 0.05) |
|  | Malaysia | 2 | 6927 | 570 | 0.03 (0.03, 0.04) |
|  | Norway | 1 | 2424 | 89 | 0.04 (0.03, 0.04) |
|  | United Kingdom | 1 | 7591 | 390 | 0.05 (0.05, 0.06) |
|  | Greece | 1 | 546 | 33 | 0.06 (0.04, 0.08) |
|  | Belgium | 2 | 2545 | 207 | 0.06 (0.05, 0.07) |
|  | Estonia | 1 | 975 | 63 | 0.06 (0.05, 0.08) |
|  | Switzerland | 1 | 206 | 14 | 0.07 (0.03, 0.10) |
|  | Taiwan | 1 | 1143 | 79 | 0.07 (0.05, 0.08) |
|  | Angola | 1 | 995 | 70 | 0.07 (0.05, 0.09) |
|  | Spain | 3 | 2862 | 233 | 0.09 (0.02, 0.16) |
|  | Thailand | 1 | 230 | 27 | 0.12 (0.08, 0.16) |
|  | China | 6 | 7829 | 1049 | 0.15 (0.10, 0.20) |
|  | Malta | 1 | 300 | 44 | 0.15 (0.11, 0.19) |
|  | South Africa | 2 | 425 | 96 | 0.16 (0.13, 0.20) |
|  | India | 3 | 6378 | 1216 | 0.17 (0.11, 0.22) |
|  | Rwanda | 1 | 922 | 157 | 0.17 (0.15, 0.19) |
|  | Guatemala | 1 | 1897 | 348 | 0.18 (0.17, 0.20) |
|  | Peru | 2 | 4709 | 1000 | 0.21 (0.20, 0.22) |
|  | Saudi Arabia | 1 | 7105 | 1492 | 0.21 (0.20, 0.22) |
|  | Brazil | 6 | 3777 | 1064 | 0.26 (0.21, 0.32) |
|  | United States of America | 7 | 6765 | 920 | 0.28 (0.19, 0.38) |
|  | Portugal | 1 | 852 | 240 | 0.28 (0.25, 0.31) |
|  | Uganda | 2 | 792 | 219 | 0.28 (0.25, 0.31) |
|  | Nepal | 2 | 921 | 263 | 0.29 (0.26, 0.31) |
|  | Japan | 2 | 386 | 120 | 0.30 (0.26, 0.35) |
|  | United Republic of Tanzania | 1 | 1116 | 337 | 0.30 (0.28, 0.33) |
|  | Nigeria | 7 | 2653 | 802 | 0.31 (0.15, 0.46) |
|  | Liberia | 1 | 183 | 59 | 0.32 (0.25, 0.39) |
|  | Nicaragua | 2 | 876 | 283 | 0.32 (0.29, 0.35) |
|  | Pakistan | 4 | 2384 | 1019 | 0.35 (0.19, 0.51) |
|  | Vietnam | 1 | 1309 | 461 | 0.35 (0.33, 0.38) |
|  | Ethiopia | 14 | 9363 | 3427 | 0.40 (0.18, 0.61) |
|  | Jordan | 1 | 303 | 124 | 0.41 (0.35, 0.46) |
|  | Mexico | 2 | 2091 | 861 | 0.41 (0.39, 0.43) |
|  | Vanuatu | 1 | 187 | 79 | 0.42 (0.35, 0.49) |
|  | Egypt | 1 | 1857 | 818 | 0.44 (0.42, 0.46) |
|  | Kenya | 3 | 893 | 386 | 0.46 (0.26, 0.65) |
|  | Mozambique | 1 | 869 | 396 | 0.46 (0.42, 0.49) |
|  | Turkey | 5 | 2235 | 612 | 0.55 (-0.03, 1.13) |
|  | Solomon Islands | 1 | 242 | 136 | 0.56 (0.50, 0.62) |
|  | Malawi | 1 | 292 | 172 | 0.59 (0.53, 0.65) |
|  | Iran | 6 | 3025 | 1935 | 0.61 (0.49, 0.73) |
|  | Gambia | 1 | 136 | 84 | 0.62 (0.54, 0.70) |
|  | Zimbabwe | 2 | 2884 | 1840 | 0.64 (0.62, 0.66) |
|  | Bangladesh | 1 | 426 | 283 | 0.66 (0.62, 0.71) |

n= IPV events. N= group size.

*Any of the IPV types (physical, psychological or sexual) combine

**Figure S1. Studies meeting each quality item**

**Figure S2. Meta-regression of IPV prevalence according to publication year**

**
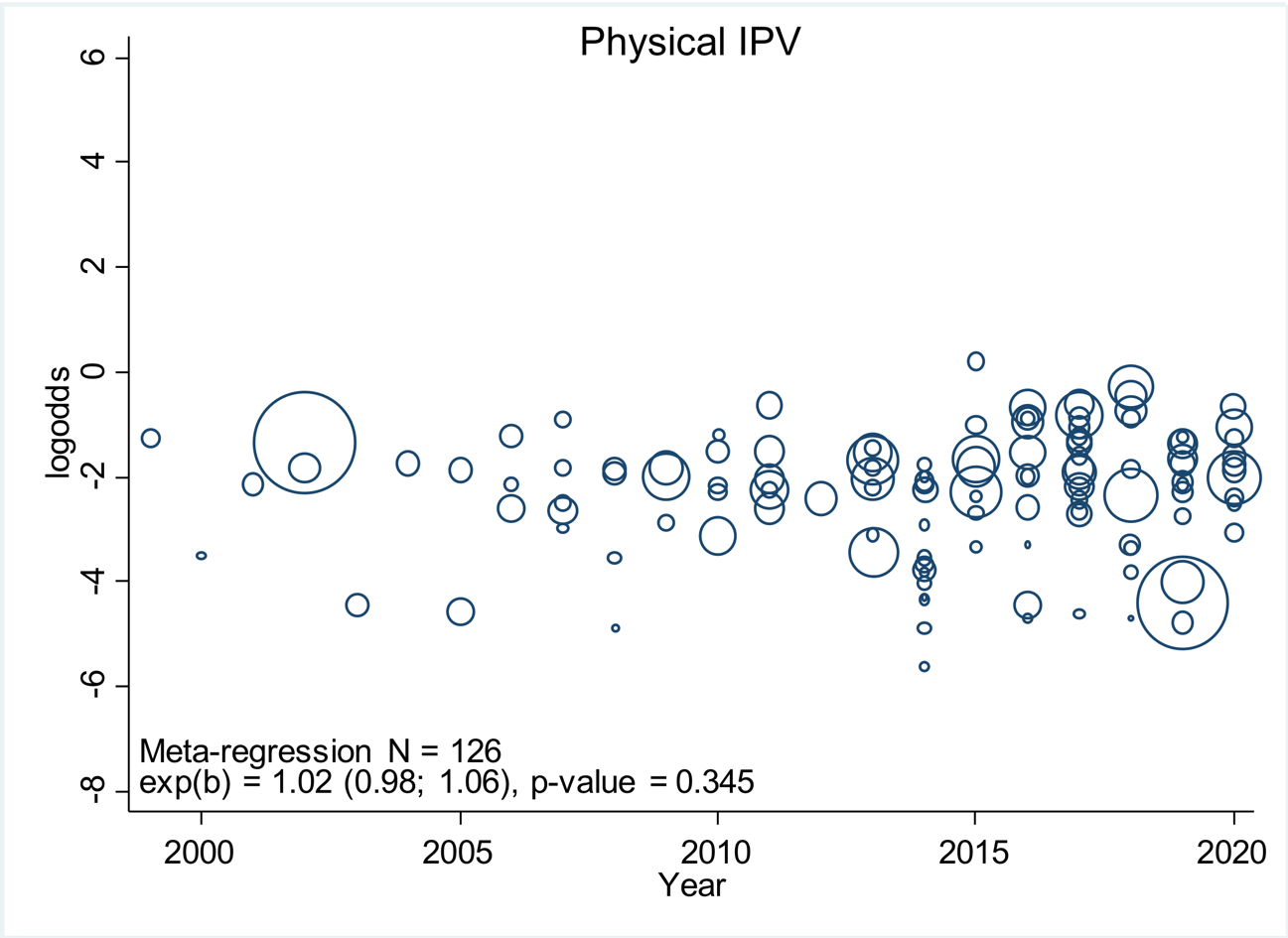
**

**
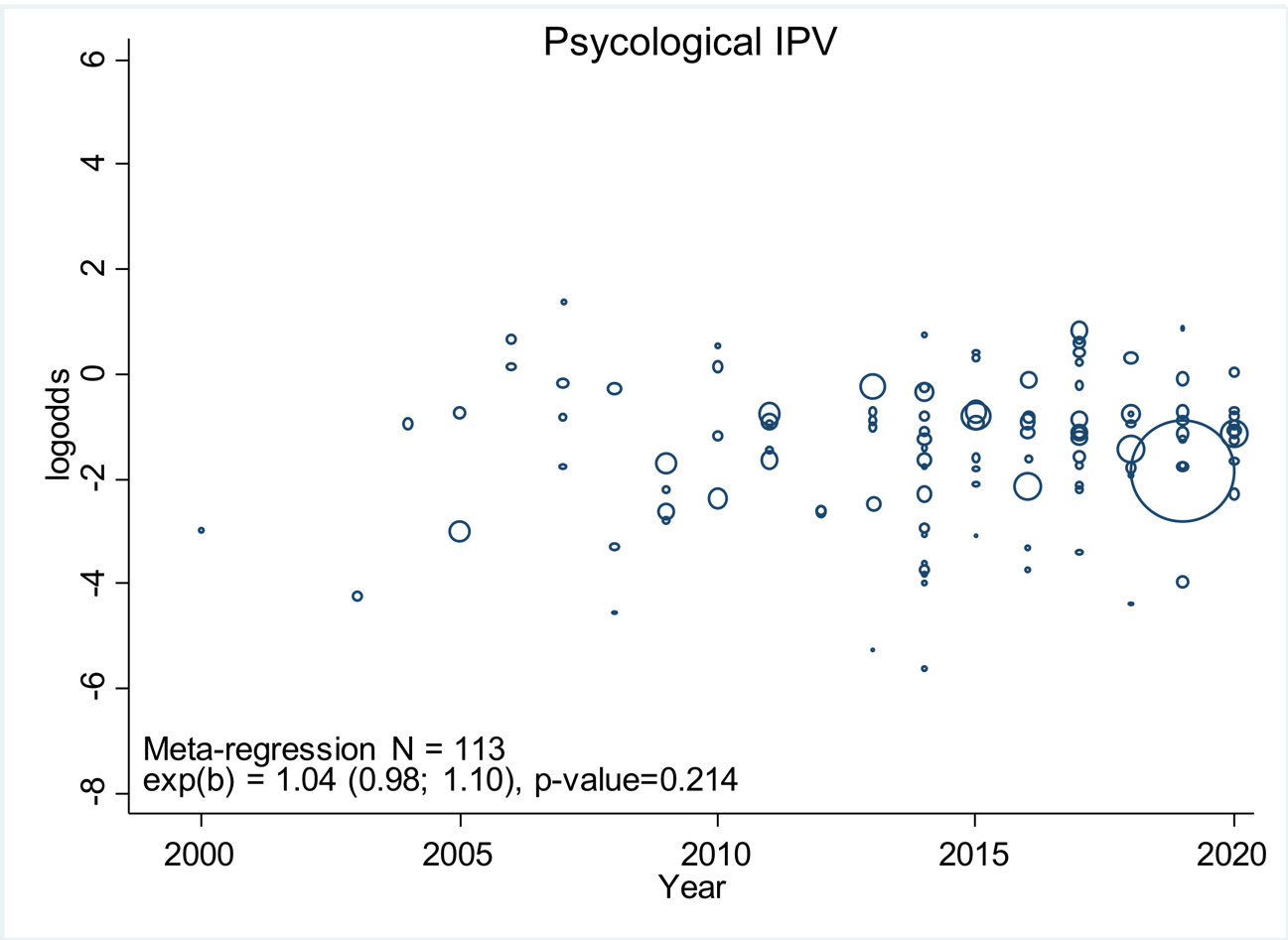
**

**
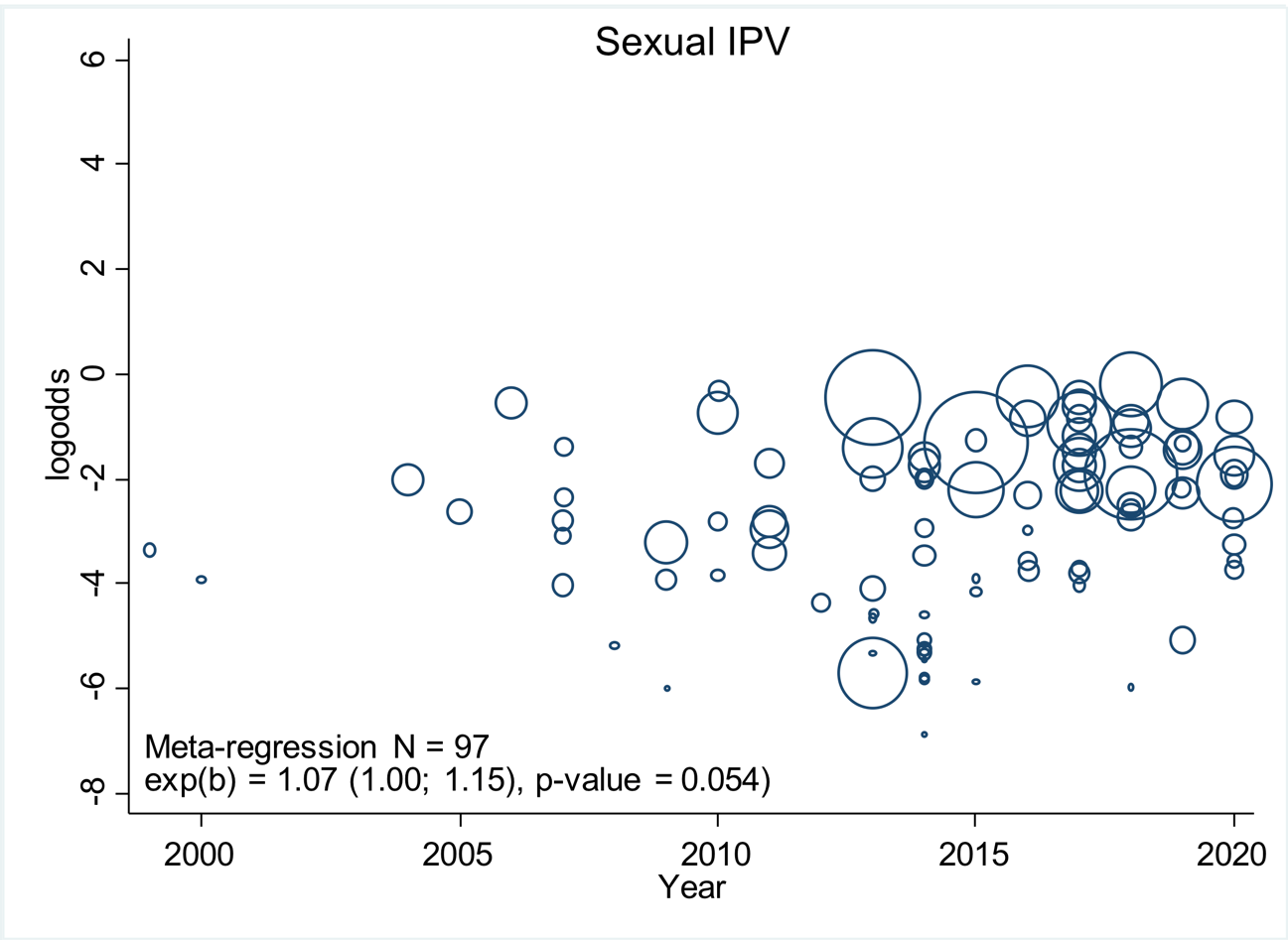
**

**
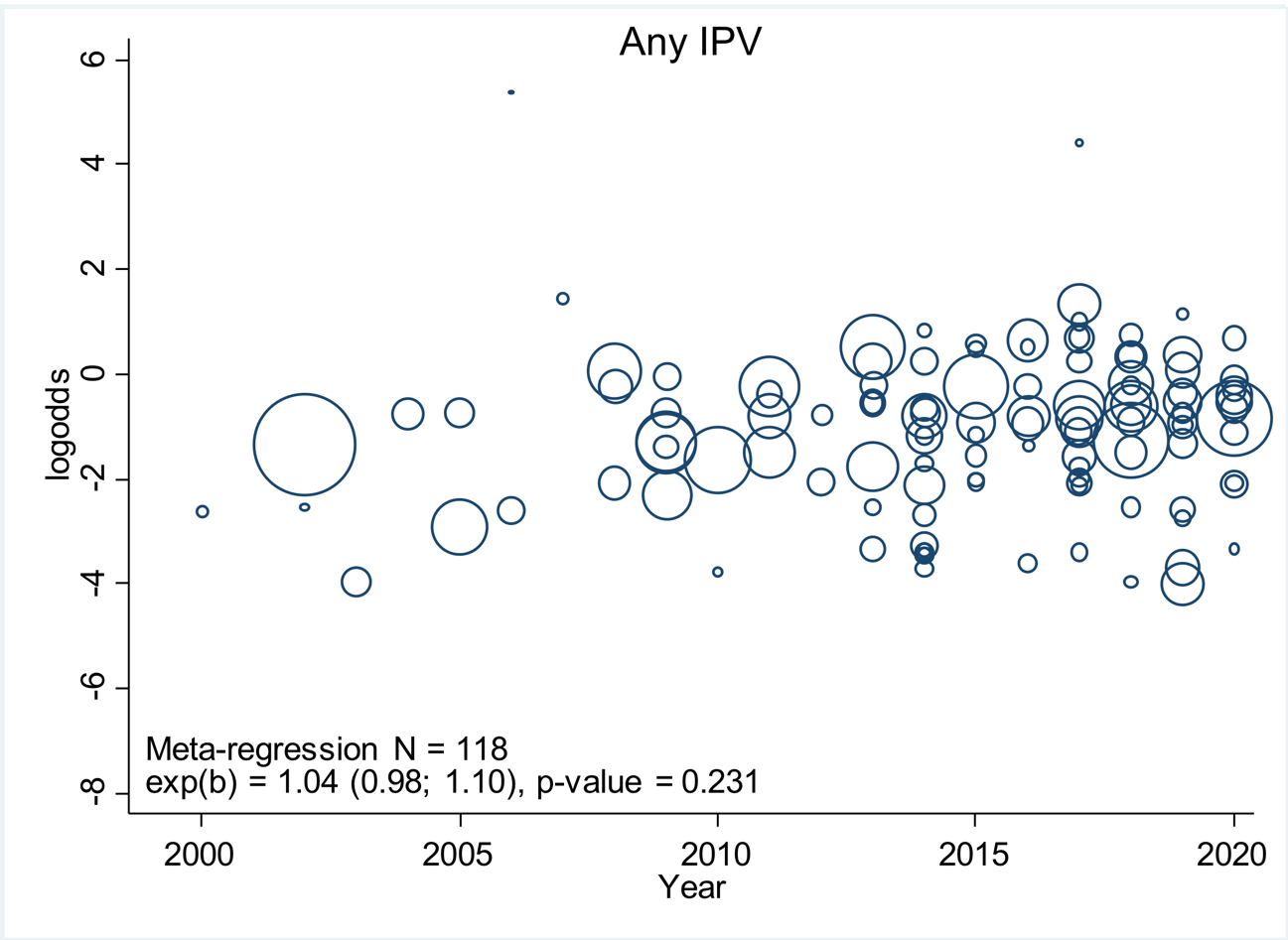
**

Association between the prevalence of IPVs (y-axis) and year of study publication (x-axis). The log odds prevalence of IPVs were modelled using a random-effects meta-regression linear model using aggregate-level data. The circle size is proportional to the precision of the prevalence estimate. The plot includes the regression slope with 95% CI and the p-value for the linear association test.

**Figure S3. Funnel plots and Egger’s tests for publication bias and small study effect**


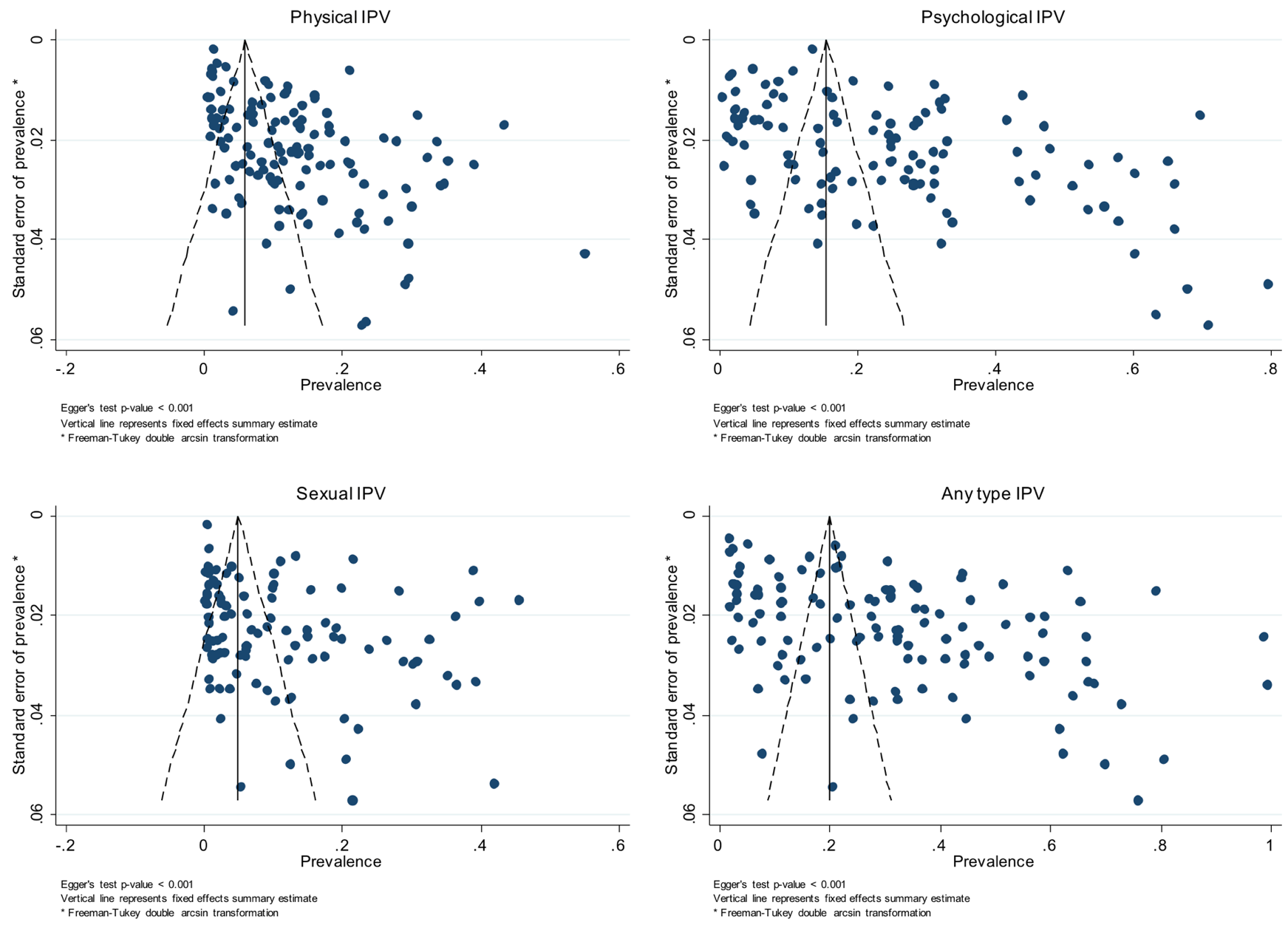

Supplement: Supplementary file 1 [file Data_Sheet_1.docx]
